# Metabolomic Profiling of Submaximal Exercise at a Standardised Relative Intensity in Healthy Adults

**DOI:** 10.3390/metabo6010009

**Published:** 2016-02-26

**Authors:** Ali Muhsen Ali, Mia Burleigh, Evangelia Daskalaki, Tong Zhang, Chris Easton, David G. Watson

**Affiliations:** 1 Strathclyde Institute of Pharmacy and Biomedical Sciences, University of Strathclyde, The John Arbuthnott Building, 161 Cathedral Street, Glasgow G4 0RE, UK; ali.muhsen.ali@strath.ac.uk (A.M.A.); evangelia.daskalaki@strath.ac.uk (E.D.); Tong.Zhang@glasgow.ac.uk (T.Z.); 2 College of Science, Thi-Qar University, Nassiriya, Thi-Qar, Iraq; 3 Institute for Clinical Exercise and Health Science, University of the West of Scotland, Hamilton ML3 0JB, UK; miacburleigh@outlook.com (M.B.); chris.easton@uws.ac.uk (C.E.)

**Keywords:** exercise metabolomics, high resolution mass spectrometry, purines, acylcarnitines, VO_2max_

## Abstract

Ten physically active subjects underwent two cycling exercise trials. In the first, aerobic capacity (VO_2max_) was determined and the second was a 45 min submaximal exercise test. Urine samples were collected separately the day before (day 1) , the day of (day 2), and the day after (day 3) the submaximal exercise test (12 samples per subject). Metabolomic profiling of the samples was carried out using hydrophilic interaction chromatography (HILIC) coupled to an Orbitrap Exactive mass spectrometer. Data were extracted, database searched and then subjected to principle components (PCA) and orthogonal partial least squares (OPLSDA) modelling. The best results were obtained from pre-treating the data by normalising the metabolites to their mean output on days 1 and 2 of the trial. This allowed PCA to separate the day 2 first void samples (D2S1) from the day 2 post-exercise samples (D2S3) PCA also separated the equivalent samples obtained on day 1 (D1S1 and D1S3). OPLSDA modelling separated both the D2S1 and D2S3 samples and D1S1 and D1S3 samples. The metabolites affected by the exercise samples included a range of purine metabolites and several acyl carnitines. Some metabolites were subject to diurnal variation these included bile acids and several amino acids, the variation of these metabolites was similar on day 1 and day 2 despite the exercise intervention on day 2. Using OPLS modelling it proved possible to identify a single abundant urinary metabolite provisionally identified as oxo-aminohexanoic acid (OHA) as being strongly correlated with VO_2max_ when the levels in the D2S3 samples were considered.

## 1. Introduction

Physical activity and exercise have a major impact on health [1] and can reduce the incidence of several diseases including cardiovascular disease (CVD) [2], diabetes [2], and mild-to-moderate depression [1]. The World Health Organisation has reported that CVDs are the leading cause of mortality (17.5 million deaths) followed by diabetes (1.5 million deaths) [3]. In the UK this is equates to more than 200,000 (37% of total) deaths due to CVD per annum [4]. The five leading global risk factors for death from CVD are: high blood pressure (13%), tobacco use (9%), high blood glucose (6%), physical inactivity (6%), and overweight/obesity (5%) [5]. These risks can be modified by choice of diet, moderation of alcohol use, and regular physical activity. It has been estimated that the indirect cost of physical inactivity in England is £8.2 billion per annum [6], in developed countries this is 1.5%–3% of total direct healthcare costs [7]; Scotland is one of the least physically active countries in Europe [8].

The type of exercise which is optimal for individuals of different cohorts (e.g., sedentary *vs.* already active) largely remains to be explored as do the molecular mechanisms underlying health improvement through exercise. Exercise optimisation may depend on various factors including gender, age, life-style, and body mass index (BMI). Directly investigating the effect of exercise on the human metabolome by using metabolomics could provide an insight into phenotypic responses and might allow personalised training regimes that are reflective of the initial metabolic status of each individual. [9]. Given that cardiorespiratory fitness is strongly associated with morbidity and mortality outcomes [10], assessing the effects of exercise on the human metabolome may also yield vital diagnostic and prognostic indicators for clinicians. Cardiorespiratory fitness is most commonly expressed as the maximal rate of oxygen consumption (VO_2max_) which can be determined during by measuring respiratory variables during an incremental exercise test to exhaustion. Of further interest is that VO_2max_ is also a primary determinant of endurance exercise performance [11]. 

Following on from our pilot study on the acute impact of exercise on individuals [12] we conducted a carefully controlled follow up study with 10 participants with a slightly modified exercise protocol. We utilised our well established hydrophilic interaction chromatography-mass spectrometry method to carry out the analysis [13,14,15,16]. The primary aim was to establish the effects of exercise on the human metabolome using a bout of exercise that was standardised to a relative intensity. The secondary aim was to explore potential predictive markers for VO_2max_.

## 2. Materials and Methods

### 2.1. Chemicals and Solvents

HPLC grade Acetonitrile (ACN) was purchased from Fisher Scientific (Loughborough, UK) and HPLC grade water was produced by a Direct-Q 3 Ultrapure Water System (Millipore, Watford, UK). AnalaR-grade formic acid (98%) was obtained from BDH-Merck (Poole, UK). Authentic stock standards were prepared as stated previously in the literature [15] and diluted four times with ACN before LC-MS analysis. Ammonium carbonate was purchased from Sigma-Aldrich (Poole, UK).

### 2.2. Subjects and Experimental Design

Ten healthy recreationally active adults, two female, eight male (mean ± S.D. (minimum–maximum) age 28 ± 7 (23–48) years.; stature, 167 ± 30 (165–189) cm; body mass, 68.5 ± 14.7 (54.5–107.5) kg; VO_2max_, 42.1 ± 5.4 (33.5–53.4)mL/kg^−1^/min^−1^; maximal cycling work rate (WR_max_) 134 ± 61 (155–369) W) volunteered and provided written informed consent to participate in the study, which was approved by the ethics committee of the School of Science and Sport, University of the West of Scotland. Participants recruited were staff and students from the University of the West of Scotland, Hamilton Campus and all had previously taken part in several maximal exercise tests. All procedures were conducted in accordance with the declaration of Helsinki.

**Figure 1:**
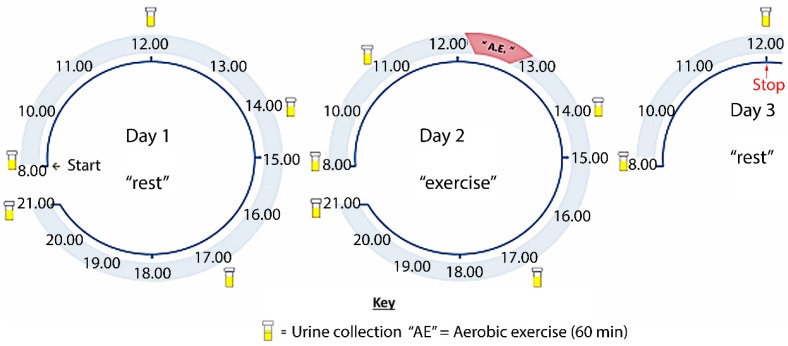
Indicative representation of urine collection schematic. The first urine sample on each day was the first pass after waking (typically 6 am–8 am).

Each participant visited the laboratory on two separate occasions to complete two cycling exercise trials. Trials were separated by at least five, but no more than 10 days. The first trial was an incremental exercise test until exhaustion in order to determine aerobic capacity (VO_2max_) and the second was a 45 min submaximal exercise test. Urine samples were collected the day before, the day of, and the day after the submaximal exercise test (Figure 1). Participants were asked not to exercise 24 h prior to the maximal test and for the duration of the urine collection period other than the submaximal trial. They were also asked to refrain from the consumption of alcohol 24 h before each test, caffeine for 6 h before testing, or consume anything other than water for 3 h before testing.

### 2.3. VO_2max_ Test

On their first visit after standard anthropological measurements, VO_2max_ and WR_Max_ were measured using a continuous graded exercise test on an electronically braked cycle ergometer (Lode Excalibur, Groningen, The Netherlands) maintaining an initial workload of 50 W. Work rate was increased in a ramp protocol at a rate of 15 W/min until the participant could no longer maintain a cadence of 50 revolutions per minute at which point the test was stopped. Throughout the test, heart rate was measured by telemetry (Polar Electro Oy, Kempele, Finland) and expired gas was measured breath by breath via indirect calorimetry (Medgraphics, Milan, Italy) and analysed for respiratory variables. The indirect calorimeter was calibrated immediately prior to each test using calibration and reference gases (calibration gas: 12% O_2_, 5% CO_2_; reference Gas: 21% O_2_, 0% CO_2_). Volume was calibrated using a 3 L syringe. Following data collection, VO_2_ data was filtered to delete values that were less than or greater than the rolling seven breath mean ± two standard deviations. All 10 participants obtained a plateau in VO_2_ (as determined by a rise in VO_2_ of <50% of the expected increase for the given WR) fulfilling the criteria for VO_2max_ [16]. Seven of the 10 participants achieved a true plateau in VO_2_ (defined as no increase or a reduction in VO_2_ despite increased WR). Although rating of perceived exertion was not recorded in the present study, all 10 particpants also achieved two of the three secondary criteria for the determination of VO_2max_. Specifically, the respiratory exchange ratio (ratio between carbon dioxide production and VO_2_) exceeded 1.10 and heart rate at the end of the maximal exercise test was within 10 beats/min of the theoretical maximum (determined by 220 − age). Smoothed data was subsequently averaged over a rolling seven breath mean and the largest value obtained was determined as the participant’s VO_2max_.

### 2.4. Submaximal Test

Participants cycled on an electronically braked cycle ergometer at light and moderate intensities for a total duration of 45 min. The exercise protocol comprised of a 5 min warm up at 50 W, 15 min at 40% of WR_Max_ (established from the initial VO_2max_ test), 15 min at 50% WR_Max_, and 10 min at 60% WR_Max_. Heart rate and expired gas were continuously monitored throughout as previously described.

### 2.5. Sample Collection and Preparation

Coded pre-labelled sterilised urine containers, Eppendorf tubes, disposable pipettes, and ziplock bags were distributed to the subjects. Subjects collected their first urine pass after waking (sample 1) on the day before, the day of, and the day after the submaximal exercise session. Typically the first sample was collected between 6 am and 8 am The time of the remaining urine collections was standardized as illustrated in Figure 1. Subjects were not restricted from urinating at any other time outside the sample windows for ethical reasons. Urine samples collected between 11 am and 17 pm were transferred to Eppendorf tubes within 5 min and deposited in a laboratory freezer at −80 °C until analysis. First pass samples and 21 pm samples were collected at home. The Eppendorf tubes were deposited inside sealed ziplock bags in the subject’s home freezer (~−20 °C) within 5 min of collection. Following completion of the study, the samples were transferred to a polystyrene freezer box surrounded by ice and ice blocks and transported to the laboratory freezer where they remained until analysis. Samples were thawed at room temperature and prepared for LC-MS analysis. Urine (200 μL) was thoroughly mixed with 800 μL of ACN, followed by centrifugation at 7840 relative centrifugal force for 5 min; 800 μL of supernatant was then transferred to a LC auto-sampler vial (Thermo Fisher, Loughborough, UK). The instrumental variation was also minimised by running the 12 samples for each subject in blocks. Since the final normalisation is subject specific, rather than being based on the full cohort this reduces the impact of technical variations in a long run. Samples are designated as follows D = day, S = sample number within a day e.g., D1S1 = day 1 sample 1. Pooled samples were interspersed throughout the run to check for technical variation. Within the blocks of 12, the samples were randomised. Four standard mixtures containing 252 standards were run also after the samples.

### 2.6. LC-MS Method

LC-MS data were acquired on an Dionex 3000 HPLC (Thermo Fisher Scientific, Hemel Hempstead, UK) coupled to an Exactive Orbitrap (Thermo Fisher Scientific) in both positive and negative mode set at 50,000 resolution (controlled by Xcalibur version 2.1.0; Thermo Fisher Scientific, Hemel Hempstead, UK). The mass scanning range was m/z 75–1200, the capillary temperature was 320 °C and the sheath and auxiliary gas flow rates were 50 and 17 arbitrary units, respectively. The separation was performed on a ZIC-pHILIC column (150 mm × 4.6 mm, 5 μm from HiChrom, Reading, UK) in binary gradient mode. The mobile phase used was 20 mM ammonium carbonate buffer (pH 9.2) and pure ACN; the flow rate was 300 μL·min^−1^. The gradient was programed as follows: 0 min 20% A 80% B to time 30 min 80% A 20% B. The injection volume was 10 μL and the sample tray temperature was controlled at 12 °C during the measurement. Samples were run in a stratified method with between subject samples placed in randomised order. MS^2^ spectra were obtained at 35 V by using an LTQ-Orbitrap under the same conditions described for the Exactive Orbitrap.

### 2.7. LC-MS Data Processing and Statistical Analysis

Raw LC-MS files were processed by using m/z Mine 2.14 [17] and the accurate masses were searched against an in house metabolite database prepared by including data from the Human Metabolome database, Lipid Maps and the Metlin database. Graphical representations, tabular features and statistical analysis (*p*-value generation) were performed in Excel (Microsoft Office 2013). The mean of the peak areas for each metabolite across the samples within each day was calculated and then the area for each metabolite was divided by the mean. This provided a subject-specific normalisation within the non-exercise and exercise days. All subsequent metabolite responses, paired *t*-test and fold changes (ratio) were conducted on the new data set. The raw data files can be accessed on the Metabolights website [18] with the identifier number MTBLS306.

### 2.8. Multivariate Analysis

SIMCA-P version 14.0 (Umetrics, Sweden) was used for multivariate analysis which included PCA, OPLS-DA and Orthogonal Partial Least Squares (OPLS). The data were centred, and Pareto scaled for PCA and OPLS-DA and OPLS in order to generate S-plots for visualisation of the components with significant influence in the dataset.

## 3. Results

### 3.1. Physiological Responses to Submaximal Exercise

The mean VO_2_ at 40%, 50%, and 60% of WR_max_ was 21.2 ± 2.5 mL/kg^−1^/min^−1^, 25.8 ± 2.5 mL/kg^−1^/min^−1^, and 30.8 ± 4.1 mL/kg^−1^/min^−1^, respectively. The mean HR during the submaximal exercise test was 115 ± 12.8 bpm, 156 ± 14 bpm, and 169 ± 13.6 bpm and 40%, 50%, and 60% or WR_max_, respectively.

### 3.2. Metabolite Profiling

Longitudinal sampling of urine provides a non-invasive method of sample collection that provides rich data sets and produces samples with greater volume than plasma, as well as containing higher concentrations of particular metabolite over the period between samplings. In contrast, even if plasma is taken frequently it will always represent an open system where taking a sample at a slightly different time might give a different answer. In this study we used a 37 h protocol (Figure 1) in order to generate an understanding of the acute effects of exercise and the duration of that response in the metabolite profiles. In addition, sampling on the pre-exercise day allowed observation of metabolite variations occurring as a diurnal rhythm. The samples were run in blocks of 12 samples for each individual with the samples randomised within each block. This method of running minimises instrumental drift since the instrument only has to remain stable over each block of 12 samples since the normalisation process for the individual within removes instrumental effects in relation to earlier or later blocks.

The output from data extraction followed by searching against the data base yielded metabolites which were identified to MSI levels 1 or 2 [19] according to either exact mass (with <3 ppm deviation) or exact mass plus retention time matching to a standard.There was no classification of the samples by PCA when the raw data was used as the basis of the model and it was also not possible to fit an OPLSDA model to the raw data was used. This was in line with our experience in our pilot study [12] where the was no separation in the PCA model when the raw data was used. In addition, in the previous study normalisation against creatinine did not make any difference to the fit of the model and it was concluded as has been observed by others that creatinine normalisation is not always effective [20].

**Figure 2:**
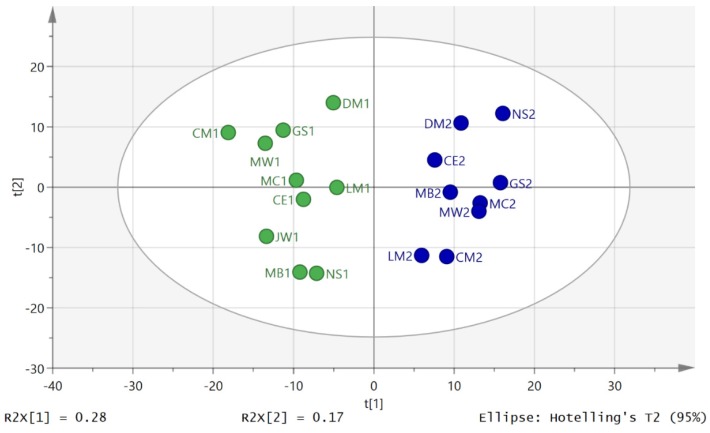
Separation of D2S3 (green) from the post exercise D2S1 samples (blue) by using PCA (R2X (cum) 0.548 Q2 (cum) 0.295, three components) following normalisation to individual metabolic output on day 2.

**Figure 3:**
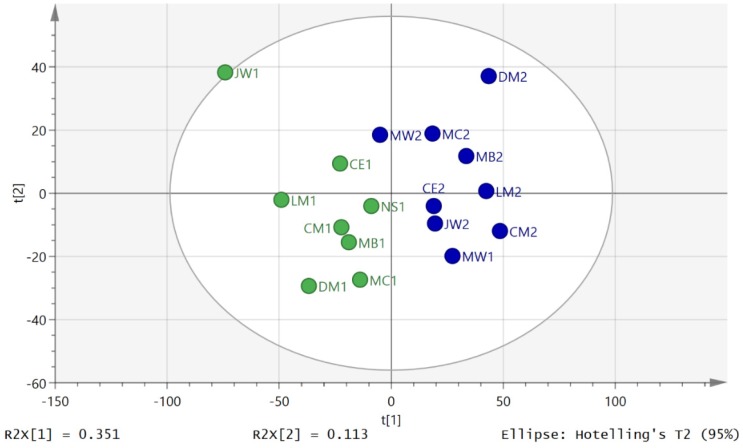
Separation of day 1 first void (D1S1 blue) and third samples (D1S3 green) by using PCA (R2X(cum) 0.536, Q2 (cum) 0.263, three components).

Thus, in order to compare metabolites on the same axis, the areas for each metabolite across all the time-points for day 1 and day 2 were averaged for each individual and then each metabolite area at each time-point was divided by the average to give the proportion contributed to the total output during the day. This approach was similar to that used in our previous study and was discussed at some length in that paper [12]. Figure 2 shows the PCA separation of 19 out 20 samples in the study for the D2S1 and D2S3 following normalisation to individual metabolic output over the five urine samples collected during day 2. One sample was excluded because there were problems with the analytical run. It is clear that there is a marked difference in metabolite profile between the two groups. The same comparison on day 1 between the first (D1S1) and third (D1S3) samples of the day, where there was no exercise intervention, gave a clear separation if one individual’s samples (GS) were excluded from the model (Figure 3). Table S1 summarises univariate comparisons for all the metabolites that are significantly changed between D2S3 in comparison with the rest of the day 2 samples and D1S3 and the rest of the day1 samples. There are a large number of changes and in order to get a clearer picture of the differences between the first and third samples on day 2 and day OPLSDA models of the data were made. Such models emphasise the importance of key variables as variables of importance (VIPs).

**Figure 4:**
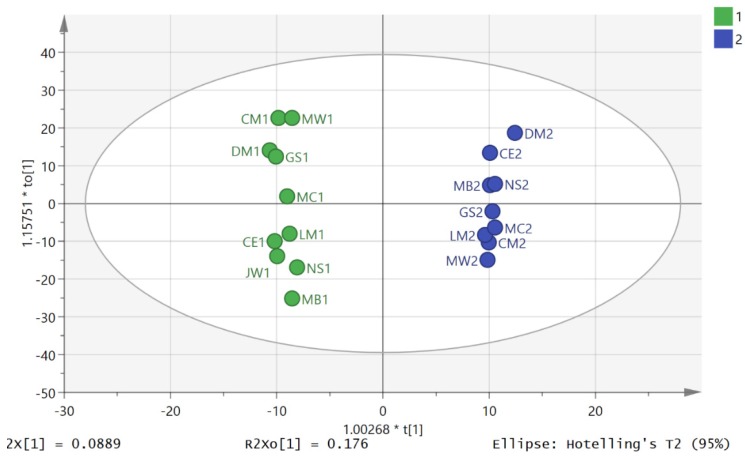
Separation of D2S1 (blue) and D2S3 (green) samples by using OPLSDA. R2X (cum) 0.35, R2Y (cum) 0.99, Q2 (cum) 0.77), three components). Based on 3078 features.

**Figure 5:**
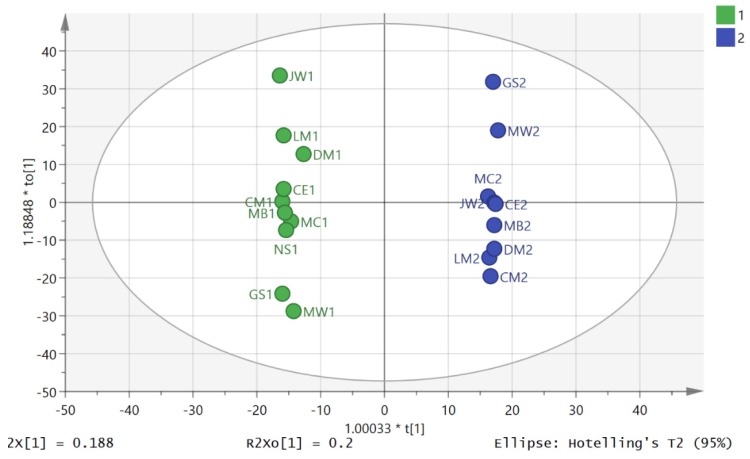
Separation of D1S1 (blue) and D1S3 (green) by OPLSDA (R2X (cum) 0.56, R2Y (cum) 0.997, Q2 (cum) 0.847, four components). Based on 3540 features.

There was a very clear separation of D2S1 and D2S3 (Figure 4) obtained by using OPLSDA for classifying D2S1/D2S3 samples. The cross validation plot indicated a strong model as can be seen in Figure S1 where all the points for the blue squares are below the blue square on the right and the plot intersects the y-axis below 0, and the CV ANOVA score for the model was 0.0029. However, a clear separation was also found between the D1S1 and D1S3 samples and the cross validation model for this was also good (Figure 5 and Figure S2), and the CV ANOVA for this model was 0.00097. Thus the composition of the metabolites in urine varies markedly throughout the day whether exercise is used as an intervention or not. Figure S3 shows an S-plot highlighting the features with the most impact (largest VIPs) on the OPLSDA model for the D2S3 and D2S1 samples and table S2 lists the highlighted metabolites. In many cases, exact identity of these features is poorly defined. However, some of the metabolites match the retention times of analytical standards or are well known urinary metabolites. Table 1 shows the metabolites which are important in the OPLSDA model shown in Figure 4 and can be identified with some confidence, the majority have *p*-values < 0.05. Application of the Benjimini-Hochberg FDR statistical test [21,22] with Q = 0.1 and including 1000 metabolites indicated that all metabolites with a *p*-value < 0.05 can be regarded as being significantly changed following exercise. Figure S4 shows an S-plot highlighting the features with the most impact on the OPLSDA model for the D1S1 and D1S3 samples and table S3 lists the metabolites with VIP values > 1.5. Some of the important changes in metabolites between the equivalent time points on day 1 and day 2 are similar.

Thus, overall, the number of changes observed as a result of exercise were fewer than in our previous smaller study but some of the changes were consistent with our previous observations particularly with regard to the impact on several purine metabolites and effects on carnitines [12]. In the current study the level of exercise was more carefully controlled than in our previous study and the subjects were in a narrow age range. The additional measurement which was made in the current study was the determination of of VO_2max_ for each of the subjects.

**Figure 6:**
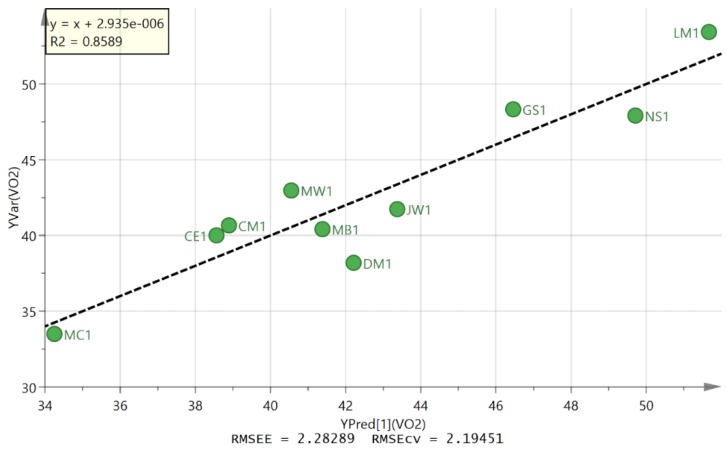
Plot of measured against predicted VO_2max_ against normalised levels of OHA.

**Figure 7:**
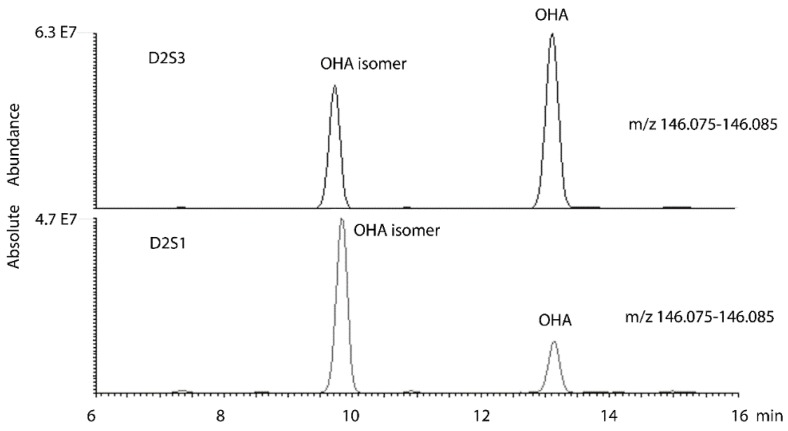
Extracted ion trace for OHA isomers in urine comparing a pre- and post-exercise sample for samples D2S1 and D2S3 for a subject with VO_2max_ 53.4.

OPLS modelling was used to predict VO_2max_ from the metabolic pattern in urine post-exercise. A weakly fitted model was built by using the D2S3 and day 2 fourth samples (D2S4) samples and then discarding variables of low importance with VIP values < 1. There seems to be no standard way to build such models and it is easy to be misled with regard to their robustness when several variables are used. It is quite surprising how easy it is for software to fit a model based on even a few variables when the number of samples is low. Eventually, by reducing the number of variables, it was possible to produce a plot of predicted against measure VO_2max_ based on a single putatively identified as being an oxoaminohexanoic acid (OHA) (Figure 6). The correlation of predicted VO_2max_ and measured VO_2max_ against normalised level of OHA gave R^2^ = 0.8589. The formula for the marker compound matched that of 11 compounds in the Metlin database [23] although there are no MS/MS spectra for any of these compounds in the database. Only seven of these compounds have an amine group which would be required for the retention time to be 13.2 min on the HILIC column as observed for the current compound. Three of the isomers occur in the lysine degradation pathway. The marker is an abundant compound in urine and two main isomers of it occur in urine with the marker compound the later running of the pair of isomers (extracted ion trace Figure 7).

The MS^2^ spectrum (Figure S5) showed losses of formic acid and water, confirming the presence of a carboxylic acid group followed by loss of a hydroxyl group but not much else. Since it seemed likely that this marker compound was in the lysine degradation pathway, another model was constructed to include all the metabolites present in the samples identified or putatively identified as being in the lysine degradation pathway according to the database search. Although it was possible to fit apparently strong models using several of these variables it was clear from the non-significant CVANOVA scores that using several variables produced over-fitting and the majority of the predictive power belonged to OHA. Table 2 shows all the predicted values for VO_2max_ based on a linear plot of the normalised levels for OHA for each individual in each sample collected on day 2. The D2S3 samples have the best predictive power although some of the other data points are predictive and this was generally in the case for the samples that did not have a strong spike in OHA post-exercise and hence are those with lower VO_2max_ values. Thus, clearly, the most important marker of VO_2max_ is the compound putatively identified as OHA. The candidate marker compound was also assessed by using an alternative method of normalisation based on absolute peak areas. It was observed that the OHA isomer remained relatively constant between D2S1 and D2S3 in comparison to OHA. The plot shown in figure S6 plots VO_2max_ against the following ratio:

(area OHA D2S3/area OHA isomer D2S3)/(area OHA D2S1/area OHA isomer D2S1)

This simpler treatment produces a plot with R^2^ = 0.654. Although the R^2^ value for this plot is not as good as the peak area normalised to individual metabolic output this gives some confidence that the observation is not an artefact of the data pre-treatment which introduces additional smoothing into the data. Thus, OHA shows some promise as a predictor of VO_2max_.

## 4. Discussion

Comparing the compounds responsible for the separation between D2S1 and D2S3, the post-exercise samples have higher levels of the purine metabolites hypoxanthine, guanine, deoxyinosine, inosine, and xanthosine. Increases in purine metabolites following exercise been observed for many years athough the focus is usually on hypoxanthine [24,25,26,27,28,29]. In our previous study we observed the same wider effect on several purine metabolites as is observed in the current data [12]. None of the above purines are significantly changed between D1S1 and D1S3 and uric acid levels decrease between D1S1 and D1S3. Thus the “purine response” obviously requires vigorous physical exercise rather than normal everyday activity to manifest. Methyl adenosine, dimethyladenosine, and dimethyl guanosine all decrease significantly between D1S1 and D1S3 but no significant changes are observed for these metabolites between D2S1 and D2S3. There are other classes of metabolites which typify the response to acute exercise. Nonanoyl carnitine, decanoyl carnitine, and ketodecanoyl carnitine all increase between D2S1 and D2S3. In contrast, several acylcarnitines are significantly decreased between D1S1 and D1S3. The increase in acyl carnitines between D2S1 and D2S3 may be related to a decreased demand for fatty acids during a short bout of exercise where there is a switch towards glycolysis and in order to maintain ATP levels under conditions of high oxygen demand. Fatty acids are removed from the mitochondria as their carnitine conjugates in order to allow acetyl CoA to enter the Krebs cycle. Panthothenic acid levels have been observed to rise following exercise previously and this is the case in the current study where there is an increase between D2S1and D2S3 whereas there is a decrease between D1S1 and D1S3. It has been proposed that increased levels of pantothenate in urine might indicate an increased requirement for CoA but it is more likely that this indicates inhibition of CoA biosynthesis which is tightly regulated by pantothenate kinase (PK) activity [30]. Acetyl CoA inhibits PK by allosteric binding and an increase in pantothenate indicates a decrease in CoA biosynthesis. Fatty acid oxidation requires an excess of CoA and a decrease in CoA levels would decrease fatty acid oxidation since it is energetically more favourable to rely on oxidation of acetyl CoA generated from glycolysis in the Krebs cycle. Lactate as the end product of glycolysis increases between D2S1 and D2S3 (Table 1) but there is no change between D1S1 and D1S3 (Table 1). Increased lactate indicates that not all the pyruvate produced by glycolysis can be used by the Krebs cycle which mainly produces energy on the form of NADH which has to oxidised via the terminal respiratory chain in order to yield ATP and thus requires oxygen. The adrenaline metabolite metanephrine is elevated post-exercise as is methoxyhydroxy phenylethyleneglycol sulfate which is also a metabolite of adrenaline. Increases in adrenaline metabolites post-exercise have been observed previously [31]. GABA levels are lower after exercise and the GABA metabolites oxobutanoate and hydroxy butyrate sulfate are elevated. Dihydroxyphenyl lactate, dihydroxybenzoate, and phenacetyl glycine acid are elevated between D2S1 and D2S3 and these are generally regarded as being products of gastrointestinal bacterial action. Elevation of microbial products has previously been reported post-exercise [9,32]. These metabolites were not changed between D1S1 and D1S3. Testosterone sulphate is elevated between D2S1 and D2S3 which might reflect and increase in testosterone levels post-exercise which has been observed previously and linked to increased anabolic metabolism [33]. Similarly, the hydrocortisone metabolite tetrahydroaldosterone glucuronide is elevated post-exercise and relects the fact the hydrocortisone is strongly involved in energy metabolism and it has been proposed that it is directly involved in nitric oxide production [34,35,36]. There are three bile acids which are important in the separation between D2S1and D2S3. Two of the bile acids are glucuronide conjugates and such conjugates are used to remove excess bile acids from the body. In the current case the levels of the conjugates fall thus indicating and possibly and increased demand for bile acids to facilitate uptake of lipids from the gut since post exercise there is an increased requirement for fatty acids. However, the same pattern is observed in the D1S1 and D1S3 comparison thus the demand for bile acids seems to be at its lowest in the early morning since none of the other time points in table S1 differ significantly from the the S3 time points and both day 1 and day 2. Several amino acids and amino acid derivatives are lowered between D2S1 and D2S3 but these changes are largely the same between D1S1 and D1S3. Exceptions include an elevation in an isomer of 5-hydroxytryptophan, possibly 3-hydroxytryptophan which was found to be elevated in our previous study [12] and carboxyethyl arginine (CEA). CEA is an advanced glycation end product that has been observed previously to occur in human urine and plasma [37,38]. This compound forms by reaction between arginine and methylglyoxal which is formed as a side product of glycolysis and it has been shown that CEA is an inhibitor of nitric oxide synthase [38] in which case its presence in the body where vassodilation is required may not be desirable.

The observation of a single metabolic marker which is apparently correlated to VO_2max_ is of great interest. A previous study aimed to find markers which could predict VO_2max_ and found that a model based on a combination of 4-ethylphenylsulfate, tryptophan, γ-tocopherol, and α-hydroxyisovalerate was predictive of VO_2max_ in a cohort of 77 subjects with R^2^ = 0.66 [39]. Although the current study is small the single OHA marker in the current case gave R^2^ = 0.8589 and thus a greater degree of correlation to VO_2max_ than the previous study. The current study can only be considered to have made a preliminary observation and a follow up trial with an independent cohort of subjects would be require to definitely prove this association. The trial could be reduced in terms of sample collection since it is the difference between the first void sample and the first post-exercise sample which is most predictive of VO_2max_ and with a high ratio of first post-exercise to first void sample indicating a high VO_2max_ as indicated by the plot shown in figure S6. Although the MW of the marker compound is small, further work is required in order to make a definitive identification since several isomers of it are known. A notable strength of the current study is that we were able to obtain valid determinations of VO_2max_ in all participants which is of vital importance in terms of assessing the predictive of any metabolic markers. This is likely due to the fact that particpants had completed several previous maximal exercise tests prior to participation in the present study. This is an important point to consider for future larger scale research work in this area.

## 5. Conclusions

The current study further validates our approach of collecting every urine sample over a period of time and then normalising each individual to their total output for each metabolite over that time. This approach allowed separation of pre- and post-exercise samples based on PCA analysis whereas without using this approach the separation was not possible. In our previous study we observed many of the changes which can be seen in the current study but there were also more changes in microbial metabolites, amino acids metabolites, acyl carnitines, and steroids [12]. The current results clearly revealed the impact of exercise on established markers for exercise such as hypoxanthine and inosine and, in addition, many other compounds were affected by exercise. In order to differentiate metabolites which vary through the day and those affected by exercise the metabolites which varied during the non-exercise day were evaluated. Some of these were the same as the metabolites changing on the non-exercise day such as bile acid metabolites but there were many metabolites which were affected only by exercise. A potential predictive marker for VO_2max_ was identified but further trials will be required to determine whether or not this is an artefact of the large number of metabolites available as variables. The approach of collecting longitudinal urine samples is simple and non-invasive and the observed output gives real sense of metabolism in action and could have a role in assessing overall fitness and diagnosing underlying disease.

## Figures and Tables

**Table 1:** Metabolites differing between D2S1 and D2S3 in comparison with D1S1 and D1S3.

Polarity	m/z	Rt	Metabolite	*p*-Value*** D2S1D2S3	Ratio D2S3D2S1	*p*-Value*** D1S1D1S3	Ratio D1S3D1S1
**Purine Metabolism**							
+	137.046	10.8	* Hypoxanthine	<0.001	3.75	0.516	0.832
+	152.057	13.1	* Guanine	0.02	2.56	na	na
−	167.021	13.5	* Urate	0.015	0.748	<0.001	0.534
−	181.037	10.8	1-Methyluric acid	0.01	0.59	0.012	0.561
+	253.093	9.2	Deoxyinosine	0.01	4.97	na	na
+	269.088	11.5	* Inosine	0.02	16.27	0.947	0.967
+	285.083	13.1	* Xanthosine	0.01	2.33	0.012	0.506
+	282.12	13.9	Methyladenosine	0.24	0.856	<0.01	0.516
+	296.136	17.8	Dimethyladenosine	0.20	2.390	<0.01	0.491
+	312.13	9.2	Dimethylguanosine	0.90	0.983	<0.003	0.562
**Neurotransmitter Metabolism**							
−	101.024	12.7	Oxobutanoate	0.030	1.212	0.690	0.953
+	104.071	14.9	* 4-Aminobutanoate	< 0.001	0.737	0.003	0.801
−	182.997	17.0	Hydroxybutyric acid sulfate	<0.001	2.172	0.519	1.133
+	198.113	16.8	* Metanephrine	0.082	3.000	0.074	0.762
−	246.992	12.8	Dihydroxy phenyl acetic acid sulfate or isomer	0.020	1.442	0.513	1.212
−	261.007	10.5	Homovanillicacid sulfate	0.142	1.397	0.857	0.932
−	263.023	8.5	Methoxyhydroxy phenylethyleneglycol sulfate	0.071	1.385	0.405	0.833
**Collagen Metabolism**							
+	173.092	13.3	Glycylproline	<0.001	0.54	0.820	1.064
+	189.123	13.6	Glycylleucine	<0.001	0.72	0.001	0.370
**Sugar Metabolism**							
−	87.0092	8.6	* Pyruvate	0.256	1.179	0.017	0.565
−	89.0242	10.2	* Lactate	0.02	2.19	0.867	1.04
−	151.061	13.6	* Xylitol or isomer	0.016	0.734	0.036	0.617
−	163.061	9.9	* Rhamnose or isomer	0.025	0.667	<0.01	0.629
	165.04	8.9	Arabinonate	0.023	0.648	<0.01	0.520
−	181.072	14.7	* Sorbitol or isomer	0.05	0.70	0.126	0.653
−	193.036	15.2	* Glucuronate or isomer	0.018	0.628	0.024	0.641
**Fatty Acid Conjugates**							
−	202.109	5.8	ǂ Hydroxyhepatonyl glycine	<0.01	1.76	0.238	1.276
+	218.139	6.0	Propanoylcarnitine	0.03	0.360	0.363	1.124
+	288.217	6.4	Octanoylcarnitine	0.5397	1.115	0.002	0.540
+	300.218	6.0	Nonanoyl carnitine	0.015	1.63	0.499	0.850
+	314.234	5.6	Decanoyl carnitine	0.01	1.56	0.391	0.825
+	330.227	6.6	Keto-decanoylcarnitine	0.032	1.61	0.499	0.850
**Steroids**							
−	367.158	3.9	Testosterone sulfate	0.04	1.57	0.319	1.23
−	539.25	6.7	Tetrahydroaldosterone glucuronide	<0.01	1.62	0.369	1.21
**Bile Acids**							
−	405.265	5.6	Dihydroxyoxocholanate	<0.01	0.44	0.078	0.512
−	407.281	6.6	Cholic acid	<0.01	0.34	<0.01	0.340
−	583.312	10.3	Cholic acid glucuronide	<0.01	0.27	< 0.01	0.170
−	583.312	11.5	Cholic acid glucuronide	<0.01	0.23	<0.01	0.170
**Amino Acids and Their Metabolites**							
−	118.051	12.0	* Threonine	<0.01	0.64	0.685	1.06
−	131.046	15.8	* Asparagine	<0.01	0.76	0.879	1.05
+	132.102	11.3	* Leucine	0.030	0.593	0.061	0.675
−	148.043	14.0	* Methionine	<0.01	0.58	0.061	0.675
+	161.128	23.0	Methyllysine	0.114	1.526	0.002	2.669
−	172.098	6.1	N-Acetylleucine	<0.01	0.65	0.010	0.590
+	175.108	9.3	** N-Acetylornithine isomer	<0.01	0.70	< 0.01	0.566
−	187.072	11.4	* N-Acetylglutamine	<0.01	0.67	< 0.01	0.628
+	221.092	6.8	** 5-Hydroxytryptophan isomer	0.06	2.24	0.530	0.952
−	214.028	14.0	Chlorotyrosine	0.04	2.09	<0.01	2.91
−	216.099	11.8	N-Acetylcitrulline	<0.01	0.60	<0.01	0.570
+	219.134	13.7	Carboxyethyllysine	<0.01	0.65	<0.01	0.467
+	247.14	15.0	Carboxyethylarginine	<0.01	2.23	0.022	0.544
**Vitamins and Co-Factors**							
+	132.077	15.4	* Creatine	0.011	0.538	0.018	0.440
+	220.118	9.4	* Pantothenate	<0.01	1.40	<0.01	0.529
−	375.13	8.1	* Riboflavin	0.013	0.66	0.032	0.500
**Microbial Metabolites**							
−	153.02	14.0	Dihydroxybenzoate	0.078	1.324	0.884	0.964
−	192.067	6.0	Phenylacetylglycine	<0.01	1.76	0.277	0.756
−	197.046	9.8	Dihydroxyphenyllactate	0.025	1.43	0.383	0.855

* Matches retention time of standard. ** Same elemental composition as the standard but different retention time. na = not detected in these samples. Matches retention time of standard. na = not detected in these samples. ǂ Same elemental composition as acetyl carnitine but retention time much earlier. *** Application of the Benjamin-Hochberg false discovery rate with Q = 0.1 and including 1000 features in the test indicated that all *p*-values < 0.05 are significant.

**Table 2:** Predicted VO_2max_ values for all samples based on normalised response for OHA.

Primary ID	Set	OAHA Normalised Level	VO_2max_	Predicted VO_2max_
105	D2S3	0.431	40	38.5
65	D2S3	0.464	40.7	38.9
51	D2S3	0.800	38.2	42.2
24	D2S3	1.225	48.3	46.5
91	D2S3	0.917	41.7	43.4
12	D2S3	1.749	53.4	51.7
118	D2S3	0.717	40.4	41.4
78	D2S3	0.000	33.5	34.3
37	D2S3	0.633	43	40.6
128	D2S3	1.554	47.9	49.7
108	D2S1	1.000	40	44.2
68	D2S1	1.069	40.7	44.9
54	D2S1	2.544	38.2	59.6
27	D2S1	1.532	48.3	49.5
94	D2S1	2.152	41.7	55.7
15	D2S1	0.335	53.4	37.6
121	D2S1	1.994	40.4	54.1
81	D2S1	1.989	33.5	54.1
40	D2S1	2.850	43	62.6
133	D2S1	0.399	47.9	38.2
107	D2S2	0.418	40	38.4
67	D2S2	0.812	40.7	42.3
53	D2S2	0.777	38.2	42.0
26	D2S2	0.634	48.3	40.6
93	D2S2	0.318	41.7	37.4
14	D2S2	1.624	53.4	50.4
120	D2S2	1.180	40.4	46.0
80	D2S2	1.419	33.5	48.4
39	D2S2	0.948	43	43.7
132	D2S2	1.320	47.9	47.4
103	D2S4	1.705	40	51.2
63	D2S4	1.384	40.7	48.0
49	D2S4	0.398	38.2	38.2
22	D2S4	0.867	48.3	42.9
89	D2S4	1.014	41.7	44.4
10	D2S4	0.937	53.4	43.6
116	D2S4	0.706	40.4	41.3
76	D2S4	0.666	33.5	40.9
35	D2S4	0.392	43	38.2
129	D2S4	1.671	47.9	50.9
101	D2S5	1.446	40	48.7
61	D2S5	1.270	40.7	46.9
47	D2S5	0.481	38.2	39.1
20	D2S5	0.743	48.3	41.7
87	D2S5	0.599	41.7	40.2
8	D2S5	0.355	53.4	37.8
114	D2S5	0.403	40.4	38.3
74	D2S5	0.926	33.5	43.5
33	D2S5	0.177	43	36.0
127	D2S5	0.057	47.9	34.8

## Supplementary Materials

The following are available online at http://www.mdpi.com/2218-1989/6/1/9/s1, Figure S1: Cross validation model for the classification of D2S1S3 by OPLSDA, Figure S2: Cross validation of the separation of D1S1 and D1S3 by OPLSDA, Figure S3: S-plot for OPLSDA model of D2S3 *vs.* D2S1 highlighting the metabolites listed in Table S1, Figure S4: S-plot for OPLSDA model of D1S3 *vs.* D1S1 highlighting the metabolites listed in Table S2, Figure S5: MS2 of oxoaminohexanoic acid obtained with a collision energy of 35 V, Figure S6: Plot of (area OHA D2S3/area OHA isomer D2S3)/(area OHA D2S1/area OHA isomer D2S1), Table S1: Comparison of D1S3 and D2S3 sample to the other samples on days 1 and 2, Table S2: Metabolites with the highest correlation to the OPLSDA model for D2S3 *vs.* D2S1, Table S3: Metabolites with the highest correlation to the OPLSDA model for D1S3 *vs.* D1S1.
