# Supplementary material for: Metabolomic Profiling of Submaximal Exercise at a Standardised Relative Intensity in Healthy Adults
Source: Metabolites. 2016 Feb 26;6(1):9. doi: 10.3390/metabo6010009 (PMC4812338; doi:10.3390/metabo6010009)
Supplement: Supplementary file 1 [file metabolites-06-00009-s001.pdf]

Supplementary Materials: Metabolomic Profiling of Pre- and Post-Exercise Urine Samples as a Predictor of VO_{2max}

Ali Muhsen Ali, Mia Burleigh, Evangelia Daskalaki, Tong Zhang, Chris Easton and David G. Watson

Table S1. Comparison of D1S3 and D2S3 sample to the other samples on days 1 and 2. na = not detected.

m/z	Rt min	Metabolite	*p*-Value D2S3 D2S1	Ratio D2S3 D2S1	P value D2S3 D2S2	Ratio D2S3 D2S2	P value D2S3 D2S4	Ratio D2S3 D2S4	P value D2S3 D2S5	Ratio D2S3 D2S5	P value D1S3 D1S1	Ratio D1S3 D2S1	P value D1S3 D1S2	Ratio D1S3 D1S2	P value D1 S3 D1S4	Ratio D1S3 D1S4	P value D1 S3 D1S5	Ratio D1S3 D1S5
137.046	10.8	Hypoxanthine	<0.001	3.75	<0.001	3.46	<0.001	5.20	<0.001	5.93	0.516	0.83	0.374	0.71	0.679	0.85	0.772	0.90
152.057	13.1	Guanine	0.018	2.56	0.009	2.96	0.002	6.01	0.006	3.52	na	na	na	na	na	na	na	na
178.072	10.8	Methyl-H2-pterin	<0.001	3.94	0.000	3.46	0.000	5.42	0.000	6.09	0.564	0.85	0.379	0.72	0.643	0.83	0.729	0.88
253.093	9.2	Deoxyinosine	0.009	4.97	na	na	na	na	na	na	na	na	na	na	na	na	na	na
165.042	13.8	Methylxanthine	0.030	0.77	0.254	1.16	0.900	0.99	0.421	1.09	0.089	0.79	0.916	1.02	0.958	0.99	0.532	0.90
165.042	8.9	Methylxanthine	0.035	0.67	0.135	1.41	0.112	1.45	0.741	0.93	0.068	0.74	0.699	1.07	0.998	1.00	0.533	0.90
167.021	13.5	Urate	0.015	0.75	0.688	0.96	0.663	0.95	0.931	0.99	<0.001	0.53	0.333	0.82	0.491	0.88	0.023	0.68
181.037	10.8	1-Methyluric acid	0.008	0.59	0.264	1.23	0.109	1.37	0.997	1.00	0.012	0.56	0.941	0.98	0.993	1.00	0.333	0.80
269.088	11.5	inosine	0.025	16.27	0.040	7.29	na	na	0.032	9.78	0.947	0.97	0.667	1.24	0.331	1.70	0.477	1.47
285.083	13.1	Xanthosine	0.011	2.33	0.002	3.98	0.002	3.93	0.012	2.44	0.012	0.51	0.350	0.74	0.290	0.71	0.183	0.64
296.135	17.7	N6,N6-Dimethyladenosine	0.197	2.39	0.104	3.66	0.047	9.09	0.036	19.29	0.002	0.49	0.192	0.77	0.508	0.90	0.350	0.85
202.109	5.8	Hydroxyhepanoylglycine	0.003	1.76	0.001	2.12	0.006	1.81	0.002	1.86	0.238	1.28	0.981	1.00	0.574	0.87	0.165	0.75
218.139	6.0	Propanoylcarnitine	0.032	0.36	0.048	10.71	0.153	3.11	0.277	2.29	0.095	0.73	0.363	1.12	0.143	0.76	0.009	0.71
248.149	12.1	Hydroxybutyrylcarnitine	0.414	1.21	0.059	1.40	0.886	1.03	0.350	0.82	0.304	0.82	0.464	0.86	0.359	0.78	0.074	0.64
274.201	6.9	Heptanoylcarnitine	0.220	1.21	0.003	1.75	0.014	1.53	0.465	1.11	0.120	0.78	0.547	0.90	0.741	0.94	0.626	0.91
314.234	5.6	Decanoyl carnitine	0.010	1.56	0.000	2.05	0.001	2.15	0.007	1.74	0.079	0.68	0.391	0.82	0.742	0.91	0.659	0.90
300.218	6.0	nonanoylcarnitine	0.015	1.63	0.003	2.10	0.003	2.08	0.057	1.45	0.064	0.60	0.499	0.85	0.789	0.93	0.682	0.89
330.227	6.6	Keto-decanoylcarnitine	0.032	1.61	0.001	2.14	0.003	2.02	0.269	1.22	<0.001	0.45	0.345	0.81	0.588	0.90	0.481	0.87
368.279	5.1	Tetradecadiencarnitine	0.158	1.72	0.004	4.73	0.004	4.24	0.041	2.18	na	na	na	na	na	na	na	na
539.25	6.7	Tetrahydroaldosterone-3-glucuronide	0.007	1.62	0.038	1.42	0.002	1.80	0.000	2.08	0.369	1.21	0.847	1.04	0.531	1.13	0.189	1.32
539.25	7.9	Tetrahydroaldosterone-3-glucuronide	0.157	1.36	0.009	1.74	0.007	1.80	0.002	1.95	0.699	1.09	0.616	0.88	0.618	1.11	0.335	1.23
413.2	4.2	Tetrahydrocorticosterone sulfate or isomer	0.124	1.37	0.412	1.19	0.023	1.72	0.032	1.65	0.672	1.10	0.980	0.99	0.198	1.34	0.053	1.56
367.158	3.9	Hydroxyandrosteone sulfate	0.037	1.57	0.806	0.95	0.171	1.40	0.147	1.44	0.319	1.23	0.453	0.87	0.208	1.28	0.031	1.50
405.265	5.6	Dihydroxy-oxo-cholanate	0.004	0.44	0.783	0.92	0.144	1.58	0.437	1.21	0.078	0.51	0.869	0.93	0.765	1.15	0.297	0.61
583.312	10.3	Cholicacidglucuronide	0.000	0.27	0.246	0.61	0.657	1.23	0.347	1.56	0.006	0.17	0.775	0.79	0.676	1.49	0.120	0.34
583.312	11.5	Cholicacidglucuronide	0.003	0.23	0.262	0.60	0.447	1.73	0.754	1.23	0.018	0.32	0.539	0.74	0.964	0.98	0.108	0.44
173.092	13.3	Glycylproline	<0.001	0.54	0.158	0.83	0.002	0.51	0.057	0.70	0.820	1.06	0.265	1.41	0.886	0.96	0.930	
187.109	13.6	Glycyl-leucine	<0.001	0.71	0.463	0.91	0.305	0.89	0.997	1.00	0.006	0.70	0.079	0.78	0.020	0.73	0.050	0.80

Table S1. *Cont.*

m/z	Rt min	Metabolite	*p*-Value D2S3 D2S1	Ratio D2S3 D2S1	P value D2S3 D2S2	Ratio D2S3 D2S2	P value D2S3 D2S4	Ratio D2S3 D2S4	P value D2S3 D2S5	Ratio D2S3 D2S5	P value D1S3 D1S1	Ratio D1S3 D2S1	P value D1S3 D1S2	Ratio D1S3 D1S2	P value D1 S3 D1S4	Ratio D1S3 D1S4	P value D1 S3 D1S5	Ratio D1S3 D1S5
87.0092	8.6	Pyruvate	0.256	1.18	0.013	1.45	0.076	1.29	0.150	1.23	0.017	0.57	0.485	0.82	0.288	0.81	0.490	0.87
89.0242	10.2	(R)-Lactate	0.018	2.19	0.106	1.58	0.234	1.35	0.078	1.63	0.867	1.04	0.161	0.57	0.137	0.71	0.708	0.91
101.024	12.7	2-Oxobutanoate	0.030	1.21	0.250	1.12	0.639	0.97	0.341	1.09	0.690	0.95	0.429	0.93	0.772	0.97	0.519	0.95
104.071	14.9	4-Aminobutanoate	0.000	0.74	0.148	0.90	0.114	0.88	0.042	0.85	0.003	0.80	0.784	0.98	0.502	0.94	0.172	0.90
182.997	17.0	hydroxybutyric acid sulfate	<0.001	2.17	0.000	1.58	0.001	1.65	0.000	2.87	0.519	1.13	0.990	1.00	0.706	0.94	0.069	1.47
198.113	16.8	L-Metanephrine	0.082	3.00	0.130	2.40	0.522	1.43	0.032	5.78	0.074	0.76	0.311	0.81	0.251	0.81	0.206	0.71
246.992	12.8	dihydroxy phenyl acetic acid sulfate or isomer	0.020	1.44	0.005	1.65	0.000	2.34	0.000	1.85	0.513	1.21	0.374	1.29	0.375	1.26	0.785	1.08
261.007	10.5	Homovanillicacidsulfate	0.142	1.40	0.148	1.34	0.022	1.72	0.014	1.83	0.857	0.93	0.529	1.29	0.275	1.65	0.306	1.60
263.023	8.5	3-Methoxy-4-hydroxyphenylethyleneglycolsulfate	0.071	1.38	0.005	1.85	0.005	1.89	0.011	1.68	0.405	0.83	0.724	1.08	0.681	1.09	0.602	1.12
111.02	15.3	Uracil	0.013	1.23	0.005	1.22	0.001	1.32	0.048	1.17	0.598	0.94	0.761	0.98	0.292	1.10	0.132	1.14
126.066	13.4	Methylcytosine	0.137	1.22	0.011	1.47	0.001	1.77	0.003	1.60	0.059	0.74	0.517	0.91	0.565	1.07	0.935	1.01
155.01	10.8	Orotate	0.032	1.34	0.618	0.92	0.390	0.88	0.147	1.26	0.061	1.40	0.947	1.01	0.407	1.16	0.016	1.53
132.077	15.4	Creatine	0.011	0.54	0.235	1.33	0.904	1.03	0.061	0.56	0.018	0.44	0.674	0.85	0.806	0.92	0.098	0.47
168.066	9.8	Pyridoxal	0.021	5.50	0.019	5.58	0.391	1.44	0.971	1.02	0.875	0.94	0.471	0.78	0.047	0.48	0.152	0.48
175.025	16.9	Ascorbate	0.062	1.27	0.309	0.84	0.399	0.89	0.195	1.23	0.404	0.80	0.954	1.02	0.121	0.66	0.231	0.63
220.118	9.4	Pantothenate	0.008	1.40	0.002	1.57	0.041	1.41	0.019	1.54	0.937	0.99	0.516	0.90	0.214	0.84	0.524	0.89
240.109	13.3	Dihydrobiopterin	0.227	1.20	0.007	1.58	0.004	1.90	0.001	1.63	0.012	0.55	0.451	0.84	0.237	0.81	0.397	0.84
252.074	14.7	Neopterin	0.016	0.61	0.449	1.18	0.341	1.24	0.828	1.06	0.046	0.45	0.801	1.11	0.732	0.88	0.784	0.90
375.13	8.1	riboflavin	0.013	0.66	0.114	1.32	0.313	1.20	0.529	0.90	0.032	0.50	0.715	0.91	0.708	0.90	0.827	1.06
118.051	12.0	Threonine	0.001	0.64	0.714	1.06	0.229	1.20	0.547	1.08	0.685	1.06	0.272	0.85	0.659	0.94	0.617	1.07
128.035	13.1	Pyrrolinehydroxycarboxylate	0.071	1.75	0.104	1.67	0.054	1.81	0.027	2.11	0.879	1.05	0.970	1.01	0.285	1.30	0.327	1.30
131.046	15.8	Asparagine	0.003	0.76	0.000	0.65	0.009	0.76	0.158	0.86	0.928	0.99	0.512	0.93	0.397	0.90	0.496	0.91
132.102	11.3	Leucine	0.030	0.59	0.533	0.84	0.267	0.74	0.039	0.55	0.061	0.67	0.361	0.83	0.303	0.81	0.485	0.88
148.043	14.0	Methionine	0.002	0.58	0.917	1.02	0.617	0.92	0.404	0.85	0.011	0.61	0.005	0.66	0.015	0.66	0.010	0.68
176.038	10.4	N-Formyl-L-methionine	0.010	0.50	0.568	1.15	0.945	0.98	0.723	0.91	0.051	0.44	0.132	0.58	0.059	0.50	0.215	0.67
206.082	5.5	N-Acetylphenylalanine	0.096	1.36	0.070	1.42	0.006	1.81	0.007	1.74	0.716	0.93	0.954	0.99	0.909	0.98	0.753	1.07
267.047	11.7	Homocystine	0.031	1.66	0.004	2.25	0.003	2.37	0.002	2.50	na	na	na	na	na	na	na	na
173.057	12.2	N-Formiminoglutamate	0.092	0.78	0.141	0.79	0.343	0.83	0.710	0.94	0.003	0.51	0.842	1.05	0.180	0.77	0.474	0.89
175.108	9.3	N-Acetylornithine	0.005	0.70	0.893	0.98	0.958	0.99	0.977	1.00	0.000	0.57	0.431	0.90	0.737	0.96	0.020	0.76
187.072	11.4	N-Acetylglutamine	0.001	0.67	0.112	0.80	0.307	0.88	0.293	0.89	0.001	0.63	0.609	0.93	0.410	0.88	0.088	0.80
188.103	14.8	5-guanidino-3-methyl-2-oxo-pentanoate	0.438	1.14	0.094	1.31	0.431	1.10	0.001	1.85	0.002	0.48	0.362	0.81	0.048	0.69	0.045	0.69
188.104	15.9	Homocitrulline	0.028	0.80	0.410	0.89	0.401	0.90	0.113	1.17	0.066	0.73	0.708	1.07	0.956	0.99	0.543	0.90
209.092	11.3	Kynurenine	0.208	0.50	0.813	0.90	0.545	1.39	0.664	1.21	0.017	0.73	0.126	0.78	0.086	0.76	0.170	0.82
216.099	11.8	N-Acetyl-citrulline	0.001	0.60	0.925	1.01	0.999	1.00	0.596	1.09	0.005	0.55	0.928	0.98	0.851	0.96	0.746	0.93
221.092	6.8	5-Hydroxy-L-tryptophan isomer	0.058	2.25	0.111	1.90	0.476	0.77	0.374	1.42	0.530	0.95	0.973	1.00	0.209	0.87	0.383	0.91

Table S1. *Cont.*

m/z	Rt min	Metabolite	*p*-Value D2S3 D2S1	Ratio D2S3 D2S1	P value D2S3 D2S2	Ratio D2S3 D2S2	P value D2S3 D2S4	Ratio D2S3 D2S4	P value D2S3 D2S5	Ratio D2S3 D2S5	P value D1S3 D1S1	Ratio D1S3 D2S1	P value D1S3 D1S2	Ratio D1S3 D1S2	P value D1 S3 D1S4	Ratio D1S3 D1S4	P value D1 S3 D1S5	Ratio D1S3 D1S5
221.092	10.7	5-Hydroxy-L-tryptophan	0.017	0.59	0.684	1.11	0.816	0.95	0.733	0.92	0.001	0.52	0.198	0.81	0.167	0.81	0.249	0.85
247.14	15.0	N2-(D-1-Carboxyethyl)-L-arginine	<0.001	2.23	0.000	2.06	0.000	1.82	0.000	3.19	0.323	0.82	0.476	0.82	0.235	0.76	0.300	0.78
245.093	13.0	N-Acetyl-D-tryptophan	0.007	0.68	0.482	0.93	0.695	1.03	0.581	0.94	0.420	0.84	0.919	1.02	0.738	1.08	0.514	1.16
247.073	9.5	5-Hydroxyindoleacetylglycine	0.109	0.53	0.495	1.42	0.589	0.80	0.551	0.77	0.474	0.82	0.619	1.15	0.984	1.01	0.366	1.30
253.051	11.1	5-L-Glutamyl-taurine	0.001	0.43	0.512	1.23	0.918	1.03	0.626	0.86	0.056	0.78	0.642	0.93	0.548	0.91	0.275	0.85
153.02	14.0	2,5-Dihydroxybenzoate	0.078	1.32	0.111	1.32	0.034	1.58	0.016	1.58	0.884	0.96	0.705	1.10	0.271	1.30	0.193	1.41
192.067	6.0	Phenylacetylglycine	0.004	1.76	0.210	1.29	0.014	1.75	0.001	2.11	0.277	0.76	0.364	0.82	0.424	0.83	0.520	0.83
197.046	9.8	3-(3,4-Dihydroxyphenyl)lactate	0.025	1.43	0.001	1.92	0.004	1.74	0.003	1.76	0.383	0.85	0.895	1.03	0.753	1.06	0.666	1.08
367.104	6.7	O-Feruloylquinate	0.054	1.74	0.366	1.30	0.416	1.25	0.524	0.81	0.018	2.41	0.816	0.92	0.199	0.70	0.423	1.24
119.035	13.4	D-Erythrose	0.001	0.67	0.967	1.00	0.792	0.98	0.558	1.07	0.334	0.87	0.010	0.66	0.329	0.87	0.105	0.76
119.035	10.9	D-Erythrose	0.027	0.73	0.924	1.02	0.259	1.25	0.423	1.16	0.032	0.63	0.017	0.59	0.069	0.68	0.021	0.61
149.046	11.1	D-Ribose	0.003	0.70	0.024	1.33	0.254	1.13	0.121	1.21	0.282	1.13	0.764	1.02	0.186	1.15	0.924	0.99
151.061	13.6	Xylitol	0.016	0.73	0.238	1.21	0.397	1.14	0.442	1.13	0.036	0.62	0.012	0.57	0.035	0.63	0.031	0.64
163.061	9.9	L-Rhamnose	0.025	0.67	0.568	1.11	0.810	0.97	0.445	0.90	0.125	0.72	0.039	0.65	0.022	0.59	0.026	0.61
165.04	8.9	L-Arabinonate	0.023	0.65	0.185	1.37	0.052	1.74	0.923	0.98	0.072	0.60	0.160	0.70	0.029	0.55	0.483	0.82
181.072	14.7	D-Sorbitol	0.050	0.70	0.598	1.08	0.800	1.04	0.172	1.26	0.126	0.65	0.070	0.60	0.047	0.55	0.144	0.67
193.036	15.2	D-Glucuronate	0.018	0.63	0.914	0.99	0.903	0.99	0.391	1.10	0.244	0.85	0.085	0.79	0.165	0.80	0.924	0.98
341.109	13.7	Sucrose	0.028	0.61	0.640	1.11	0.228	0.78	0.036	1.79	0.090	0.70	0.300	0.85	0.189	0.81	0.970	1.01
173.082	13.2	Suberic acid	0.039	0.52	0.598	0.80	0.944	0.98	0.386	0.78	0.001	0.47	0.353	0.83	0.889	1.04	0.311	1.34
199.097	11.1	Decenedioic acid	0.007	0.49	0.497	1.13	0.669	0.92	0.034	0.64	0.412	0.96	0.110	1.09	0.805	0.99	0.169	1.08
217.108	13.0	Hydroxysebacicacid	0.066	0.53	0.498	0.82	0.715	0.88	0.649	0.88	0.120	0.53	0.228	0.68	0.983	1.01	0.203	0.64

Table S2. Metabolites with the highest correlation to the OPLSDA model for D2S3 vs D2S1. Metabolites with positive p (corr) values associate with D2S1 and metabolites with negative p(corr) values associate with D2S3.

m/z	Rt	Molecular Formula	Metabolite	*p*-Value D2S3/D2S1	Ratio D2S3/D2S1	p(corr)
409.183	14.7	$C_{22}H_{28}F_2O_5$	Flumethasone	0.00001	0.24	0.89
583.312	10.3	$C_{30}H_{48}O_{11}$	Cholicacid glucuronide	0.00009	0.27	0.63
407.164	15.2	$C_{22}H_{29}O_5Cl$	Beclomethasone	0.00012	0.20	0.68
331.09	10.9	$C_{13}H_{21}N_2O_4PS$	Butamifos	0.00015	0.48	0.78
173.092	13.3	$C_7H_{12}N_2O_3$	Glycylproline	0.00015	0.54	0.75
477.147	12.8	$C_{17}H_{26}N_4O_{12}$	Glu-Thr-Asp-Asp	0.00026	0.42	0.72
291.15	10.2	$C_{19}H_{20}N_2O$	16-Epivellosimine	0.00029	0.33	0.61
461.225	9.8	$C_{19}H_{34}N_4O_9$	Glu-Leu-Thr-Thr	0.00035	0.24	0.67
233.068	11.9	$C_9H_{14}O_7$	trihomocitrate	0.00038	0.50	0.71

Table S2. *Cont.*

m/z	Rt	Molecular Formula	Metabolite	*p*-Value D2S3/D2S1	Ratio D2S3/D2S1	p(corr)
471.218	9.1	$C_{30}H_{32}O_5$	[Fv] Euchrenone a14	0.00049	0.26	0.81
173.073	12.4	$C_{10}H_{10}N_2O$	Indole-3-acetamide	0.00058	0.61	0.70
375.152	4.0	$C_{26}H_{20}N_2O$	XE991	0.00073	0.10	0.65
407.281	6.6	$C_{24}H_{40}O_5$	[ST trihydrox] 3Alpha,7Alpha,12Alpha-trihydroxy-5Beta-cholan-24-oic acid	0.00079	0.34	0.74
118.051	12.0	$C_4H_9NO_3$	L-Threonine	0.00079	0.64	0.71
281.114	11.9	$C_{13}H_{18}N_2O_5$	Thr-Tyr	0.00082	0.39	0.68
322.09	11.2	$C_{11}H_{21}N_3O_4S_2$	Met-Ala-Cys	0.00106	0.27	0.65
291.114	9.7	$C_{18}H_{16}N_2O_2$	INF271	0.00109	0.55	0.64
220.029	11.1	$C_7H_{11}NO_5S$	S-(3-oxo-3-carboxy-n-propyl)cysteine	0.00111	0.21	0.63
288.124	12.1	$C_{16}H_{19}NO_4$	3'-Demethoxypiplartine	0.00117	0.26	0.71
377.146	13.2	$C_{17}H_{22}N_4O_6$	Trp-Ser-Ser	0.00122	0.29	0.71
302.136	12.6	$C_{12}H_{21}N_3O_6$	Nicotianamine	0.00122	0.47	0.67
413.219	4.9	$C_{21}H_{34}O_8$	[FA methyl(20:2)] methyl 5-hydroperoxy-6,8,9,11-bisepidioxy-12,14-eicosadienoate	0.00126	0.59	0.64
216.099	11.8	$C_8H_{15}N_3O_4$	N-Acetyl-L-citrulline	0.00132	0.60	0.63
334.055	10.8	$C_9H_{14}N_5O_7P$	Dihydroneopterin phosphate	0.00146	0.39	0.64
172.097	10.9	$C_8H_{13}NO_3$	N-Butyryl-L-homoserine lactone	0.00147	0.60	0.67
389.179	8.3	$C_{25}H_{27}N_2Cl$	Meclizine	0.00161	0.50	0.67
148.043	14.0	$C_5H_{11}NO_2S$	L-Methionine	0.00165	0.58	0.63
399.124	12.9	$C_{25}H_{20}O_5$	[Fv methoxy,methox] (S)-2,3-Dihydro-7-methoxy-2-[2-(4-methoxyphenyl)-5-benzofuranyl]-4H-1-benzopyran-4-one	0.00166	0.45	0.63
215.104	9.3	$C_9H_{16}N_2O_4$	gamma-Glutamyl-gamma-aminobutyraldehyde	0.00180	0.64	0.68
247.129	13.4	$C_{10}H_{18}N_2O_5$	Glu-Val	0.00187	0.40	0.62
583.312	11.5	$C_{30}H_{48}O_{11}$	Cholicacidglucuronide	0.00253	0.23	0.50
321.089	14.1	$C_{10}H_{18}N_4O_6S$	Asn-Cys-Ser	0.00282	0.39	0.59
421.171	12.0	$C_{19}H_{26}N_4O_7$	Ala-Phe-Ala-Asp	0.00373	0.31	0.54
449.203	10.4	$C_{21}H_{30}N_4O_7$	Ala-Phe-Val-Asp	0.00400	0.32	0.74
388.168	10.2	$C_{20}H_{24}FN_3O_4$	Balofloxacin	0.00534	0.14	0.50
379.161	11.7	$C_{17}H_{24}N_4O_6$	Ala-Gln-Tyr	0.00542	0.35	0.63
191.103	9.8	$C_7H_{14}N_2O_4$	meso-2,6-Diaminoheptanedioate	0.00647	0.30	0.57
214.028	9.7	$C_9H_{10}C_lNO_3$	3-Chlorotyrosine	0.00697	0.23	0.59
142.051	9.6	$C_6H_9NO_3$	Vinylacetylglycine	0.00755	0.56	0.67
301.129	14.9	$C_{15}H_{18}N_4O_3$	Phe-His	0.00904	0.31	0.59
254.096	14.5	$C_{13}H_{18}ClNO_2$	Hydroxybupropion	0.00917	0.49	0.63

Table S2. *Cont.*

m/z	Rt	Molecular Formula	Metabolite	*p*-Value D2S3/D2S1	Ratio D2S3/D2S1	p(corr)
261.144	5.6	$C_{11}H_{20}N_2O_5$	Glu-Leu	0.01123	0.31	0.60
322.113	9.0	$C_{13}H_{23}NO_4S_2$	S-Glutaryldihydrolipoamide	0.01264	0.47	0.58
403.161	11.1	$C_{19}H_{24}N_4O_6$	Glu-Ala-Trp	0.01338	0.26	0.65
258.108	15.7	$C_{10}H_{17}N_3O_5$	Gly-Pro-Ser	0.02019	0.25	0.50
421.228	4.4	$C_{20}H_{38}O_7S$	1,4-Bis(2-ethylhexyl) sulfosuccinate	0.02783	0.33	0.51
218.139	6.0	$C_{10}H_{19}NO_4$	O-Propanoylcarnitine	0.03150	0.36	0.45
147.066	11.1	$C_6H_{12}O_4$	[FA methyl,hydroxy(5:0)] 3R-methyl-3,5-dihydroxy-pentanoic acid	0.03859	0.61	0.55
297.181	4.6	$C_{13}H_{22}N_5O_3$	diphthamide	0.04240	0.47	0.46
386.145	15.5	$C_{18}H_{21}N_5O_5$	benzyladenine-7-N-glucoside	0.04767	0.34	0.57
379.168	12.0	$C_{22}H_{24}N_2O_4$	Vomicine	0.05349	0.34	0.58
469.16	4.5	$C_{19}H_{26}N_4O_{10}$	Asp-Ser-Ser-Tyr	0.09988	0.29	0.47
434.214	7.9	$C_{19}H_{29}N_7O_5$	Phe-Asn-Arg	0.14187	0.38	0.51
247.14	15.0	$C_9H_{18}N_4O_4$	N2-(D-1-Carboxyethyl)-L-arginine	0.00001	2.23	−0.89
178.072	10.8	$C_7H_7N_5O$	6-methyl-H2-pterin	0.00007	3.94	−0.90
182.997	17.0	$C_4H_8SO_6$	hydroxybutyric acid sulfate	0.00008	2.17	−0.85
250.105	10.8	$C_8H_{15}N_3O_6$	Gly-Ser-Ser	0.00010	3.43	−0.87
137.046	10.8	$C_5H_4N_4O$	Hypoxanthine	0.00011	3.75	−0.89
190.072	9.6	$C_7H_{13}NO_5$	2-amino-3,7-dideoxy-D-threo-hept-6-ulosonate	0.00073	1.42	−0.77
217.083	10.9	$C_8H_{14}N_2O_5$	L-Ala-L-Glu	0.00096	1.76	−0.73
240.102	9.1	$C_{15}H_{13}NO_2$	N-Hydroxy-2-acetamidofluorene	0.00169	3.88	−0.66
140.082	8.9	$C_6H_9N_3O$	L-Histidinal	0.00201	4.27	−0.67
240.001	15.7	$C_6H_{11}NO_5S_2$	3-Mercaptolactate-cysteinedisulfide	0.00246	1.53	−0.68
202.109	5.8	$C_9H_{18}NO_4$	O-Acetylcarnitine	0.00260	1.76	−0.69
192.067	6.0	$C_{10}H_{11}NO_3$	Phenylacetylglycine	0.00432	1.76	−0.63
172.097	13.5	$C_8H_{13}NO_3$	N-Butyryl-L-homoserine lactone	0.00546	3.77	−0.58
555.245	8.8	$C_{28}H_{36}N_4O_8$	Asp-Leu-Phe-Tyr	0.00753	1.48	−0.70
253.093	9.2	$C_{10}H_{12}N_4O_4$	Deoxyinosine	0.00884	4.97	−0.76
314.234	5.6	$C_{17}H_{33}NO_4$	[FA (10:0)] O-decanoyl-R-carnitine	0.00975	1.56	−0.65
285.083	13.1	$C_{10}H_{12}N_4O_6$	Xanthosine	0.01137	2.33	−0.61
391.019	17.0	$C_{18}H_{17}O_5Br$	[Fv hydroxy,trimethox] 3'-Bromo-6'-hydroxy-2',4,4'-trimethoxychalcone	0.01425	1.79	−0.57
231.009	4.1	$C_3H_{11}N_3O_5PS$	N-Phosphohypotaurocyamine	0.01992	1.59	−0.64
168.066	8.0	$C_8H_9NO_3$	Pyridoxal	0.02063	5.50	−0.54
541.265	9.6	$C_{24}H_{34}N_{10}O_5$	His-Leu-His-His	0.02189	1.84	−0.63

Table S2. *Cont.*

m/z	Rt	Molecular Formula	Metabolite	*p*-Value D2S3/D2S1	Ratio D2S3/D2S1	p(corr)
269.088	11.5	$C_9H_{16}O_9$	inosine	0.02489	16.27	−0.55
197.046	9.8	$C_9H_{10}O_5$	3-(3,4-Dihydroxyphenyl)lactate	0.02514	1.43	−0.63
498.27	6.1	$C_{26}H_{35}N_5O_5$	Lys-Phe-Phe-Gly	0.03237	1.75	−0.61
132.066	14.2	$C_5H_9NO_3$	hydroxyproline	0.03886	18.28	−0.46
161.038	19.7	$C_7H_{11}O_2Cl$	[FA (7:0)] 7-chloro-2E-heptenoic acid	0.04844	1.90	−0.51
221.092	6.8	$C_{11}H_{12}N_2O_3$	5-Hydroxy-L-tryptophan	0.05752	2.25	−0.46
450.136	4.5	$C_{20}H_{25}N_3O_7S$	(1R)-Hydroxy-(2R)-glutathionyl-1,2-dihydronaphthalene	0.05860	1.98	−0.56
198.113	16.8	$C_{10}H_{15}NO_3$	L-Metanephrine	0.08210	3.00	−0.39
395.071	11.3	$C_{12}H_{20}N_4O_7S_2$	Asp-Cys-Cys-Gly	0.09705	2.66	−0.62
420.223	4.5	$C_{20}H_{29}N_5O_5$	Lys-Trp-Ser	0.11143	2.04	−0.58
173.092	14.9	$C_7H_{12}N_2O_3$	Glycylproline	0.11562	5.58	−0.38
170.081	12.4	$C_8H_{11}NO_3$	Pyridoxine	0.19003	2.05	−0.32

Table S3. Metabolites with the highest correlation to the OPLSDA model for D1S3 vs D1S1. Metabolites with positive p (corr) values associate with D1S1 and metabolites with negative p(corr) values associate with D1S3.

m/z	Rt	Molecular Formula	Metabolite	*p*-Value	Ratio D1S3/D1S1	p(corr)
334.126	18.0	$C_{12}H_{21}N_3O_8$	N4-(Acetyl-beta-D-glucosaminyl)asparagine	0.00098	19.59	0.79
277.002	11.5	$C_9H_{10}SO_8$	trihydroxyphenylpropionic acid sulfate or isomer	0.02027	22.15	0.84
330.118	10.8	$C_{14}H_{19}NO_8$	Dopamineglucuronide	0.02707	4.62	0.50
379.122	5.4	$C_{19}H_{24}O_6S$	[ST methoxy,hydroxy(3:0)] 2-methoxy-3-hydroxy-estra-1,3,5(10)-trien-17-one 3-sulfate	0.01368	6.81	0.73
409.136	8.7	$C_{17}H_{22}N_4O_8$	Asn-Asp-Tyr	0.05651	5.74	0.58
319.045	4.2	$C_{15}H_{12}O_8$	Dihydromyricetin	0.04583	2.89	0.67
367.066	15.7	$C_{10}H_{15}N_4O_9P$	1-(5'-Phosphoribosyl)-5-formamido-4-imidazolecarboxamide	0.00296	6.45	0.74
240.101	10.8	$C_{15}H_{13}NO_2$	N-Hydroxy-2-acetamidofluorene	0.00378	3.09	0.57
346.056	11.2	$C_{10}H_{14}N_5O_7P$	AMP	0.06766	6.58	0.56
490.216	5.1	$C_{19}H_{33}N_5O_{10}$	Glu-Lys-Thr-Asp	0.05040	3.00	0.51
234.077	9.5	$C_{12}H_{11}NO_4$	2-Hydroxy-6-oxo-(2'-aminophenyl)-hexa-2,4-dienoate	0.00034	4.54	0.80
597.366	5.0	$C_{33}H_{48}N_4O_6$	L-Urobilinogen	0.00696	6.71	0.69
343.118	7.9	$C_{19}H_{18}O_6$	[Fv Methyl,trimethox] 3,4-Methylenedioxy-2',4',6'-trimethoxychalcone	0.01677	5.06	0.63
367.104	6.4	$C_{17}H_{20}O_9$	O-Feruloylquinate	0.01823	2.41	0.70
188.003	5.1	$C_6H_7NO_4S$	2-Pyridyl hydroxymethane sulfonic acid	0.00313	2.56	0.73
200.175	12.1	$C_{10}H_{21}N_3O$	Diethylcarbamazine	0.01824	4.84	0.62
381.18	14.2	$C_{22}H_{24}N_2O_4$	Vomicine	0.00890	2.89	0.60

Table S3. *Cont.*

m/z	Rt	Molecular Formula	Metabolite	*p*-Value	Ratio D1S3/D1S1	p(corr)
240.109	10.8	$C_9H_{13}N_5O_3$	Dihydrobiopterin	0.00080	2.72	0.76
359.204	24.0	$C_{14}H_{26}N_6O_5$	Pro-Ser-Arg	0.00625	4.80	0.67
302.134	16.5	$C_{12}H_{19}N_3O_6$	Ala-Asp-Pro	0.00152	4.57	0.80
277.003	14.4	$C_9H_{10}SO_8$	trihydroxyphenylpropionic acid sulfate or isomer	0.06894	3.20	0.51
385.144	6.4	$C_{13}H_{24}N_2O_{11}$	Macrozamin	0.00710	4.28	0.72
298.093	8.9	$C_{13}H_{17}NO_7$	p-aminobenzoate-β-D-glucopyranosyl ester	0.01663	2.27	0.57
218.049	4.5	$C_8H_{11}NO_4S$	Tyramine-O-sulfate	0.02041	2.32	0.66
295.087	7.8	$C_{12}H_{16}N_4O_3S$	5'-methylthiotubercidin	0.02420	3.72	0.39
302.087	13.7	$C_{12}H_{15}NO_8$	4-Nitrophenol-alpha-D-galactopyranoside	0.00129	2.55	0.70
261.091	4.3	$C_{10}H_{16}N_2O_4S$	d-biotin d-sulfoxide	0.03123	1.94	0.63
176.001	5.6	$C_5H_5NSO_4$	hydroxypyridine sulfate	0.07406	2.56	0.46
381.137	5.3	$C_{22}H_{23}ClN_2O_2$	Loratadine	0.00094	2.69	0.73
595.35	5.1	$C_{33}H_{46}N_4O_6$	L-Urobilin	0.00315	2.99	0.59
275.092	10.9	$C_{15}H_{16}O_5$	Lactucin	0.04030	1.91	0.54
315.159	12.9	$C_{19}H_{22}O_4$	[PR] 2,3-Didehydrogibberellin A10	0.00014	2.12	0.74
234.077	12.7	$C_{12}H_{11}NO_4$	2-Hydroxy-6-oxo-(2'-aminophenyl)-hexa-2,4-dienoate	0.00010	2.98	0.81
270.061	11.7	$C_{11}H_{13}NO_7$	hydroxypyridine glucuronide	0.09258	2.11	0.57
163.052	8.8	$C_7H_{11}O_2Cl$	[FA (7:0)] 7-chloro-2E-heptenoic acid	0.09062	2.00	0.51
446.152	16.4	$C_{16}H_{25}N_5O_{10}$	Ala-Asp-Asp-Gln	0.04092	3.77	0.53
156.077	17.2	$C_6H_9N_3O_2$	L-Histidine	0.00012	3.54	0.85
161.128	23.0	$C_7H_{16}N_2O_2$	N6-Methyl-L-lysine	0.00168	2.67	0.73
593.334	5.3	$C_{32}H_{48}O_{10}$	Debromoaplysiatoxin	0.01163	2.41	0.53
173.987	5.6	$C_5H_5NSO_4$	hydroxypyridine sulfate	0.03154	2.07	0.65
594.337	5.2	$C_{26}H_{43}N_9O_7$	Arg-Lys-Gln-Tyr	0.01468	2.29	0.50
411.122	6.0	$C_{20}H_{25}O_7Cl$	Eupachlorin	0.01506	4.31	0.62
156.077	15.8	$C_6H_9N_3O_2$	L-Histidine	0.00002	2.63	0.87
327.072	13.9	$C_{14}H_{16}O_9$	2-Succinyl-5-enolpyruvyl-6-hydroxy-3-cyclohexene-1-carboxylate	0.04770	2.09	0.46
221.081	6.0	$C_{12}H_{12}O_4$	2,6-Dioxo-6-phenylhexanoate	0.04714	1.97	0.56
273.008	5.2	$C_{10}H_{10}SO_7$	ferulic acid sulfate or isomer	0.21485	1.87	0.49
154.062	15.7	$C_6H_9N_3O_2$	L-Histidine	0.00166	2.73	0.72
196.026	13.5	$C_8H_7NO_5$	3-Hydroxy-2-methylpyridine-4,5-dicarboxylate	0.05050	2.03	0.49
399.144	16.6	$C_{22}H_{22}O_7$	Deoxypodophyllotoxin	0.00169	3.17	0.76

Table S3. *Cont.*

m/z	Rt	Molecular Formula	Metabolite	*p*-Value	Ratio D1S3/D1S1	p(corr)
168.03	7.8	$C_7H_7NO_4$	L-2,3-Dihydrodipicolinate	0.02339	1.98	0.60
214.027	14.0	$C_9H_{10}ClNO_3$	3-Chlorotyrosine	0.00298	2.91	0.71
156.077	19.1	$C_6H_9N_3O_2$	L-Histidine	0.00007	1.99	0.78
169.051	7.9	$C_8H_{10}O_4$	3,4-Dihydroxyphenylethyleneglycol	0.01602	2.17	0.63
260.053	12.7	$C_6H_{14}NO_8P$	D-Glucosamine 6-phosphate	0.00029	2.14	0.78
154.062	17.2	$C_6H_9N_3O_2$	L-Histidine	0.00222	2.35	0.70
154.063	20.4	$C_6H_9N_3O_2$	L-Histidine	0.00026	2.10	0.79
207.077	4.9	$C_{10}H_{12}N_2O_3$	L-Kynurenine	0.01873	1.58	0.54
217.018	4.3	$C_8H_{10}SO_5$	phenylethylene glycol sulfate	0.02391	2.08	0.64
309.076	5.1	$C_{18}H_{12}O_5$	[Fv Methyl(9:1)] 3',4'-Methylenedioxy-[2'',3'':7,8]furanoflavanone	0.26933	2.04	0.39
78.9185	13.6	BrH	Br-	0.00050	2.28	0.77
175.119	25.3	$C_6H_{14}N_4O_2$	L-Arginine	0.02416	1.83	0.56
180.089	17.1	$C_7H_{10}N_5O$	N1-hydroxyethyladenine	0.00002	2.11	0.82
174.057	9.3	$C_{10}H_9NO_2$	Indole-3-acetate	0.01749	1.79	0.54
215.033	14.1	$C_5H_{13}O_7P$	2-C-Methyl-D-erythritol 4-phosphate	0.01439	2.71	0.62
241.129	14.6	$C_{10}H_{16}N_4O_3$	Homocarnosine	0.05667	2.67	0.46
230.997	6.9	$C_8H_8SO_6$	hydroxyphenyl acetic acid sulfate or isomer	0.03176	2.29	0.59
415.094	16.0	$C_{15}H_{20}N_4O_8S$	O-Carbamoyl-deacetylcephalosporin C	0.01044	2.53	0.58
234.077	16.3	$C_{12}H_{11}NO_4$	2-Hydroxy-6-oxo-(2'-aminophenyl)-hexa-2,4-dienoate	0.00694	1.51	0.61
208.062	14.3	$C_{10}H_{11}NO_4$	N-Benzyloxycarbonylglycine	0.00070	1.75	0.69
116.035	14.1	$C_4H_7NO_3$	L-2-Amino-3-oxobutanoic acid	0.00273	1.51	0.67
357.169	15.0	$C_{21}H_{24}O_5$	[Fv Trihydroxy,trimethy] 2',4',4''-Trihydroxy-3',6'',6''-trimethylpyrano[2'',3'':6',5']dihydrochalcone	0.03435	2.01	0.52
170.092	13.5	$C_7H_{11}N_3O_2$	N(pi)-Methyl-L-histidine	0.00040	1.69	0.74
340.154	15.6	$C_{20}H_{21}NO_4$	(S)-Canadine	0.00114	1.58	0.70
114.021	14.7	$C_4H_5NO_3$	Maleamate	0.02136	1.55	0.55
285.119	14.8	$C_{11}H_{16}N_4O_5$	Coformycin	0.00560	2.27	0.62
234.077	18.0	$C_{12}H_{11}NO_4$	2-Hydroxy-6-oxo-(2'-aminophenyl)-hexa-2,4-dienoate	0.00615	1.69	0.63
128.035	16.1	$C_5H_7NO_3$	L-1-Pyrroline-3-hydroxy-5-carboxylate	0.00398	1.47	0.60
323.146	22.9	$C_{12}H_{24}N_2O_8$	Procollagen 5-(D-galactosyloxy)-L-lysine	0.05039	2.99	0.53
386.159	12.1	$C_{21}H_{23}NO_6$	Polycarpine	0.00156	1.54	0.69
180.086	17.1	$C_6H_{13}NO_5$	D-Glucosamine	0.00479	1.61	0.61

Table S3. *Cont.*

m/z	Rt	Molecular Formula	Metabolite	*p*-Value	Ratio D1S3/D1S1	p(corr)
157.061	11.9	$C_6H_8N_2O_3$	4-Imidazolone-5-propanoate	0.00015	1.38	0.75
249.149	12.5	$C_{15}H_{20}O_3$	[PR] 1,2-Dihydrosantonin	0.28230	2.11	0.40
267.138	6.8	$C_{18}H_{18}O_2$	[ST hydroxy(5:0)] 3-hydroxy-estra-1,3,5(10),6,8-pentaen-17-one	0.00244	0.48	−0.66
275.151	7.4	$C_{14}H_{20}N_4O_2$	N-(4-Guanidinobutyl)-4-hydroxycinnamide	0.00465	0.39	−0.61
287.196	4.7	$C_{14}H_{26}N_2O_4$	N-Acetyl-leucyl-leucine	0.00226	0.53	−0.68
450.176	6.5	$C_{15}H_{27}N_7O_7S$	Arg-Asp-Cys-Gly	0.00170	0.51	−0.71
153.031	11.3	$C_6H_6N_2O_3$	Imidazol-5-yl-pyruvate	0.00346	0.46	−0.64
174.087	15.0	$C_6H_{11}N_3O_3$	5-Guanidino-2-oxopentanoate	0.00038	0.53	−0.73
494.239	5.0	$C_{25}H_{35}NO_9$	Ryanodine	0.00242	0.48	−0.68
262.136	5.7	$C_{14}H_{19}N_3S$	Methapyrilene	0.00094	0.56	−0.72
172.097	9.0	$C_8H_{13}NO_3$	N-Butyryl-L-homoserine lactone	0.00021	0.50	−0.74
245.113	9.2	$C_{10}H_{16}N_2O_5$	Glu-Pro	0.00047	0.56	−0.74
451.166	8.0	$C_{20}H_{28}N_4O_6S$	Ala-Cys-Pro-Tyr	0.00380	0.39	−0.63
111.009	18.5	$C_5H_4O_3$	2-Furoate	0.00253	0.48	−0.64
172.073	15.0	$C_6H_{11}N_3O_3$	5-Guanidino-2-oxopentanoate	0.00020	0.45	−0.76
500.239	4.5	$C_{19}H_{33}N_9O_5S$	Arg-Met-Gly-His	0.00248	0.52	−0.68
538.232	5.0	$C_{24}H_{35}N_5O_7S$	Gln-Met-Pro-Tyr	0.00016	0.39	−0.78
237.09	8.3	$C_8H_{16}N_2O_4S$	Met-Ser	0.00581	0.52	−0.61
522.197	5.6	$C_{18}H_{31}N_7O_9S$	Glu-Asp-Cys-Arg	0.00234	0.40	−0.69
210.062	5.0	$C_7H_7O_3N_5$	uric acid acetonitrile adduct	0.00074	0.52	−0.71
422.203	7.8	$C_{19}H_{27}N_5O_6$	Phe-Gln-Gln	0.00230	0.53	−0.65
492.224	5.0	$C_{18}H_{33}N_7O_7S$	Ala-Met-Asp-Arg	0.00130	0.50	−0.70
241.012	14.9	$C_6H_{11}O_8P$	D-myo-Inositol 1,2-cyclic phosphate	0.00031	0.57	−0.73
434.217	7.5	$C_{23}H_{31}NO_7$	Mycophenolate mofetil	0.00049	0.43	−0.70
102.066	16.8	$C_3H_7N_3O$	N-acetylguanidine	0.00001	0.46	−0.83
156.078	10.0	$C_6H_{10}N_3O_2$	N3-hydroxyethylcytosine	0.00034	0.52	−0.74
448.203	6.1	$C_{17}H_{29}N_5O_9$	Ala-Lys-Asp-Asp	0.00023	0.48	−0.76
234.134	12.9	$C_{10}H_{19}NO_5$	Hydroxypropionylcarnitine	0.00219	0.52	−0.66
176.103	16.2	$C_6H_{13}N_3O_3$	L-Citrulline	0.00044	0.59	−0.77
219.134	13.7	$C_9H_{18}N_2O_4$	N2-(D-1-Carboxyethyl)-L-lysine	0.00014	0.47	−0.77
138.091	22.4	$C_8H_{11}NO$	Tyramine	0.00118	0.53	−0.70
290.102	4.9	$C_{15}H_{15}NO_5$	Acronycidine	0.00020	0.49	−0.77

Table S3. *Cont.*

m/z	Rt	Molecular Formula	Metabolite	*p*-Value	Ratio D1S3/D1S1	p(corr)
336.217	6.2	$C_{19}H_{29}NO_4$	Ankorine	0.00013	0.56	−0.77
189.123	13.6	$C_8H_{16}N_2O_3$	Glycyl-leucine	0.00013	0.54	−0.77
207.114	20.0	$C_{11}H_{14}N_2O_2$	Phenylethylmalonamide	0.00001	0.56	−0.85
374.254	7.2	$C_{19}H_{35}NO_6$	Dodecanedioylcarnitine	0.00001	0.51	−0.84
173.009	18.4	$C_6H_6O_6$	cis-Aconitate	0.00267	0.40	−0.64
129.019	18.5	$C_5H_6O_4$	Mesaconate	0.00237	0.42	−0.65
278.198	5.2	$C_{14}H_{23}N_5O$	EHNA	0.00034	0.41	−0.78
146.081	9.8	$C_6H_{11}NO_3$	[FA oxo,amino(6:0)] 3-oxo-5S-amino-hexanoic acid	0.00013	0.54	−0.77
493.208	8.0	$C_{25}H_{34}O_{10}$	Glaucarubinone	0.00082	0.20	−0.56
288.171	4.7	$C_{16}H_{21}N_3O_2$	Zolmitriptan	0.00195	0.51	−0.68
203.139	14.6	$C_9H_{18}N_2O_3$	Leu-Ala	0.00016	0.58	−0.79
476.199	5.5	$C_{18}H_{29}N_5O_{10}$	Asn-Leu-Asp-Asp	0.00415	0.48	−0.62
258.11	15.2	$C_8H_{20}NO_6P$	sn-glycero-3-Phosphocholine	0.00108	0.55	−0.70
500.247	4.5	$C_{20}H_{33}N_7O_8$	Asp-Lys-Thr-His	0.00106	0.52	−0.72
334.203	6.2	$C_{19}H_{29}NO_4$	Ankorine	0.00579	0.48	−0.59
220.029	15.6	$C_7H_{11}NO_5S$	S-(3-oxo-3-carboxy-n-propyl)cysteine	0.00138	0.53	−0.70
218.114	11.8	$C_8H_{15}N_3O_4$	N-Acetyl-L-citrulline	0.00001	0.57	−0.83
490.207	5.0	$C_{18}H_{31}N_7O_7S$	Arg-Asp-Cys-Pro	0.00156	0.51	−0.71
178.107	16.2	$C_7H_{15}NO_4$	validamine	0.00060	0.56	−0.75
221.092	10.7	$C_{11}H_{12}N_2O_3$	5-Hydroxy-L-tryptophan	0.00066	0.52	−0.74
165.075	13.4	$C_6H_{12}O_5$	L-Rhamnose	0.00042	0.52	−0.74
233.114	10.9	$C_9H_{16}N_2O_5$	N2-Succinyl-L-ornithine	0.00196	0.52	−0.64
288.217	6.4	$C_{15}H_{29}NO_4$	L-Octanoylcarnitine	0.00161	0.54	−0.69
283.129	5.5	$C_{13}H_{18}N_2O_5$	Thr-Tyr	0.00135	0.49	−0.71
219.081	14.9	$C_8H_{16}N_2O_3S$	Met-Ala	0.00027	0.52	−0.72
187.108	9.8	$C_8H_{14}N_2O_3$	Ala-Pro	0.00004	0.52	−0.81
295.132	6.0	$C_{19}H_{18}O_3$	(2-Butylbenzofuran-3-yl)(4-hydroxyphenyl)ketone	0.00117	0.59	−0.69
145.014	16.2	$C_5H_6O_5$	2-Oxoglutarate	0.00348	0.47	−0.63
480.187	7.9	$C_{16}H_{29}N_7O_8S$	Arg-Asp-Cys-Ser	0.00143	0.44	−0.70
220.118	13.2	$C_9H_{17}NO_5$	Pantothenate	0.00146	0.53	−0.69
153.066	11.9	$C_7H_8N_2O_2$	N1-Methyl-2-pyridone-5-carboxamide	0.00096	0.52	−0.68
257.113	9.6	$C_{11}H_{16}N_2O_5$	1-(beta-D-Ribofuranosyl)-1,4-dihydronicotinamide	0.00116	0.56	−0.71

Table S3. *Cont.*

m/z	Rt	Molecular Formula	Metabolite	*p*-Value	Ratio D1S3/D1S1	p(corr)
450.235	5.5	$C_{21}H_{31}N_5O_6$	Ala-Phe-Val-Asn	0.00007	0.45	−0.78
248.113	14.1	$C_{10}H_{17}NO_6$	Linamarin	0.00032	0.51	−0.75
350.16	7.2	$C_{18}H_{23}NO_6$	Riddelline	0.00378	0.52	−0.64
217.129	15.6	$C_8H_{16}N_4O_3$	N-acetyl-(L)-arginine	0.00001	0.54	−0.85
247.092	11.3	$C_9H_{14}N_2O_6$	5-6-Dihydrouridine	0.00016	0.55	−0.76
368.155	13.4	$C_{14}H_{25}NO_{10}$	N-Acetyl-6-O-L-fucosyl-D-glucosamine	0.02483	0.33	−0.51
287.148	12.1	$C_{10}H_{20}N_6O_4$	Asn-Arg	0.00005	0.31	−0.79
204.134	12.7	$C_8H_{17}N_3O_3$	Lys-Gly	0.00142	0.52	−0.70
118.086	14.6	$C_5H_{11}NO_2$	L-Valine	0.00124	0.52	−0.73
403.192	5.0	$C_{26}H_{28}O_4$	[Fv] Boesenbergin A	0.00029	0.51	−0.73
245.15	8.8	$C_{11}H_{20}N_2O_4$	N-hexenoylglutamine	0.00007	0.48	−0.83
464.192	5.4	$C_{16}H_{29}N_7O_7S$	Ala-Asp-Cys-Arg	0.00097	0.48	−0.73
386.255	8.3	$C_{21}H_{31}N_5O_2$	Buspirone	0.00015	0.41	−0.75
310.113	14.2	$C_{11}H_{19}NO_9$	N-Acetylneuraminate	0.00006	0.58	−0.78
163.108	9.8	$C_6H_{14}N_2O_3$	N6-Hydroxy-L-lysine	0.00004	0.44	−0.83
346.124	14.8	$C_{15}H_{24}NO_4PS$	Isofenphos	0.00081	0.53	−0.71
288.171	8.8	$C_{16}H_{21}N_3O_2$	Zolmitriptan	0.00010	0.46	−0.76
313.062	11.2	$C_{10}H_{20}O_7P_2$	[PR] Geranyl pyrophosphate	0.00019	0.33	−0.74
288.125	12.0	$C_{16}H_{19}NO_4$	3'-Demethoxypiplartine	0.00067	0.27	−0.64
167.021	13.5	$C_5H_4N_4O_3$	Urate	0.00019	0.53	−0.76
379.163	9.3	$C_{17}H_{24}N_4O_6$	Ala-Gln-Tyr	0.00302	0.48	−0.64
253.143	5.2	$C_{14}H_{20}O_4$	ubiquinol-1	0.00007	0.53	−0.79
253.123	7.7	$C_{17}H_{16}O_2$	cis-Hinokiresinol	0.00448	0.41	−0.64
417.211	7.2	$C_{16}H_{30}N_6O_7$	Ala-Lys-Asn-Ser	0.00094	0.40	−0.70
341.101	14.6	$C_{19}H_{16}O_6$	Psorospermin	0.00568	0.44	−0.60
175.072	12.4	$C_6H_{12}N_2O_4$	Ala-Ser	0.00056	0.54	−0.71
431.177	4.0	$C_{17}H_{26}N_4O_9$	Glu-Ala-Asp-Pro	0.00143	0.48	−0.70
384.274	5.7	$C_{21}H_{37}NO_5$	3-Hydroxy-5, 8-tetradecadiencarnitine	0.00018	0.50	−0.76
326.087	11.1	$C_{14}H_{15}NO_8$	Pancratistatin	0.00045	0.52	−0.74
230.116	13.4	$C_9H_{16}N_5Cl$	Propazine	0.00007	0.45	−0.79
402.285	6.4	$C_{21}H_{39}NO_6$	[SP] Myriocin	0.00046	0.47	−0.74
175.108	9.3	$C_7H_{14}N_2O_3$	N-Acetylornithine	0.00016	0.57	−0.77

Table S3. *Cont.*

m/z	Rt	Molecular Formula	Metabolite	*p*-Value	Ratio D1S3/D1S1	p(corr)
131.118	20.5	$C_6H_{14}N_2O$	N-Acetylputrescine	0.00331	0.55	−0.66
120.066	12.0	$C_4H_9NO_3$	L-Threonine	0.00009	0.42	−0.80
327.139	14.2	$C_{20}H_{19}FO_3$	2,2,4-Trimethyl-3-(4-fluorophenyl)-2H-1-benzopyran-7-ol acetate	0.00007	0.55	−0.78
205.035	17.8	$C_7H_{10}O_7$	2-Hydroxybutane-1,2,4-tricarboxylate	0.00138	0.49	−0.73
487.256	8.1	$C_{25}H_{36}N_4O_6$	Ile-Pro-Pro-Tyr	0.00153	0.25	−0.68
389.182	8.6	$C_{19}H_{26}N_4O_5$	Phe-Gln-Pro	0.00025	0.42	−0.74
227.127	22.4	$C_{12}H_{18}O_4$	[FA oxo,hydroxy(5:1/5:0)] (1S,2R)-3-oxo-2-(5'-hydroxy-2'Z-pentenyl)-cyclopentaneacetic acid	0.00840	0.53	−0.60
336.158	7.7	$C_{13}H_{25}N_3O_5S$	Leu-Thr-Cys	0.00430	0.55	−0.63
274.092	14.1	$C_{12}H_{19}NO_2S_2$	Brugine	0.00003	0.48	−0.80
216.099	11.8	$C_8H_{15}N_3O_4$	N-Acetyl-L-citrulline	0.00501	0.55	−0.62
431.197	7.5	$C_{18}H_{32}N_4O_6S$	Cys-Leu-Thr-Pro	0.00444	0.47	−0.62
290.089	10.2	$C_{11}H_{17}NO_8$	2,7-Anhydro-alpha-N-acetylneuraminic acid	0.01615	0.41	−0.59
157.992	7.5	$C_5H_5NO_3S$	3-pyridinesulfonate	0.01051	0.42	−0.56
472.25	4.3	$C_{19}H_{33}N_7O_7$	Asn-Lys-Asn-Pro	0.00001	0.45	−0.83
235.092	12.8	$C_8H_{14}N_2O_6$	Glu-Ser	0.00007	0.52	−0.80
166.05	5.1	$C_8H_7NO_3$	Formylanthranilate	0.00117	0.57	−0.69
260.089	15.6	$C_9H_{15}N_3O_6$	Ala-Asp-Gly	0.01361	0.44	−0.60
330.19	5.0	$C_{16}H_{27}NO_6$	Europine	0.00011	0.49	−0.77
259.092	13.7	$C_{10}H_{14}N_2O_6$	(1-Ribosylimidazole)-4-acetate	0.00006	0.49	−0.80
494.193	5.7	$C_{18}H_{31}N_5O_9S$	Asp-Met-Thr-Gln	0.00334	0.20	−0.71
231.17	13.7	$C_{11}H_{22}N_2O_3$	Leu-Val	0.00024	0.46	−0.77
344.182	5.0	$C_{15}H_{25}N_3O_6$	Leu-Asp-Pro	0.00001	0.43	−0.83
247.132	8.4	$C_{15}H_{18}O_3$	[PR] alpha-Santonin	0.00003	0.46	−0.80
282.12	13.9	$C_{11}H_{15}N_5O_4$	1-Methyladenosine	0.00042	0.52	−0.77
420.189	5.1	$C_{19}H_{25}N_5O_6$	Thr-Trp-Asn	0.00007	0.44	−0.81
272.172	5.3	$C_{11}H_{21}N_5O_3$	Pro-Arg	0.00012	0.41	−0.78
164.074	13.3	$C_6H_{13}NO_2S$	S-Methyl-L-methionine	0.00105	0.49	−0.72
290.088	12.5	$C_{11}H_{17}NO_8$	2,7-Anhydro-alpha-N-acetylneuraminic acid	0.00080	0.50	−0.69
283.123	13.9	$C_{20}H_{14}N_2$	2,3-Diphenyl-3-(2-pyridinyl)acrylonitrile	0.00045	0.51	−0.76
217.118	9.3	$C_9H_{16}N_2O_4$	gamma-Glutamyl-gamma-aminobutyraldehyde	0.00006	0.51	−0.81
170.092	10.4	$C_7H_{11}N_3O_2$	N(pi)-Methyl-L-histidine	0.00002	0.44	−0.83

Table S3. *Cont.*

m/z	Rt	Molecular Formula	Metabolite	*p*-Value	Ratio D1S3/D1S1	p(corr)
512.287	5.0	$C_{27}H_{37}N_5O_5$	Ala-Lys-Phe-Phe	0.00004	0.41	−0.82
360.24	5.2	$C_{19}H_{29}N_5O_2$	Loxtidine	0.00237	0.57	−0.66
244.154	8.8	$C_{12}H_{21}NO_4$	Tiglylcarnitine	0.00004	0.43	−0.82
221.096	12.9	$C_8H_{16}N_2O_3S$	Met-Ala	0.00294	0.52	−0.66
462.199	6.4	$C_{21}H_{27}N_5O_7$	Ala-Trp-Ala-Asp	0.00014	0.42	−0.81
312.13	9.2	$C_{12}H_{17}N_5O_5$	1-7-Dimethylguanosine	0.00255	0.56	−0.66
206.102	15.9	$C_8H_{15}NO_5$	N-Acetyl-D-fucosamine	0.00054	0.45	−0.75
450.23	5.0	$C_{16}H_{31}N_7O_8$	Arg-Thr-Ser-Ser	0.00001	0.46	−0.84
261.145	8.9	$C_{11}H_{20}N_2O_5$	Glu-Leu	0.00006	0.43	−0.82
294.1	13.8	$C_{14}H_{16}N_3O_2Cl$	Triadimefon	0.00410	0.48	−0.61
184.061	5.1	$C_8H_9NO_4$	4-Pyridoxate	0.00054	0.53	−0.73
245.149	7.3	$C_{11}H_{20}N_2O_4$	N-hexenoylglutamine	0.00080	0.51	−0.71
232.129	10.9	$C_9H_{17}N_3O_4$	Ala-Ala-Ala	0.00077	0.46	−0.70
320.062	20.4	$C_{11}H_{15}NO_{10}$	beta-Citryl-L-glutamic acid	0.14503	0.52	−0.57
188.057	14.9	$C_7H_{11}NO_5$	N-Acetyl-L-glutamate	0.00005	0.40	−0.78
333.115	11.1	$C_{13}H_{21}N_4O_2ClS$	Tos-Arg-CH2Cl	0.00362	0.46	−0.62
333.105	7.8	$C_{11}H_{18}N_4O_8$	Asn-Asp-Ser	0.00723	0.50	−0.58
526.265	5.5	$C_{27}H_{35}N_5O_6$	Asn-Phe-Phe-Val	0.00549	0.53	−0.62
357.213	14.3	$C_{16}H_{28}N_4O_5$	Leu-Gln-Pro	0.00013	0.47	−0.77
222.09	15.7	$C_8H_{16}NO_4P$	D,L-cyclohexanephosphinothricin	0.00097	0.55	−0.69
392.155	12.5	$C_{16}H_{25}NO_{10}$	Proacaciberin	0.00015	0.39	−0.78
234.145	9.3	$C_9H_{19}N_3O_4$	Lys-Ser	0.00008	0.45	−0.81
296.136	17.8	$C_{12}H_{17}N_5O_4$	N6,N6-Dimethyladenosine	0.00195	0.49	−0.69
530.333	6.3	$C_{28}H_{43}N_5O_5$	Ile-Leu-Trp-Val	0.00106	0.50	−0.70
192.106	17.8	$C_8H_{17}NO_2S$	trihomomethionine	0.00549	0.48	−0.60
478.192	9.8	$C_{21}H_{27}N_5O_8$	Ala-Trp-Asp-Ser	0.00201	0.34	−0.64
301.077	8.5	$C_{10}H_{12}N_4O_7$	urate-3-ribonucleoside	0.00745	0.28	−0.66
531.336	6.3	$C_{21}H_{42}N_{10}O_6$	Arg-Leu-Ser-Arg	0.00055	0.44	−0.73
400.27	6.3	$C_{21}H_{39}NO_6$	[SP] Myriocin	0.00400	0.39	−0.60
790.56	3.8	$C_{42}H_{80}NO_{10}P$	PS(18:0/18:1(9Z))	0.00432	0.53	−0.63
331.175	11.7	$C_{16}H_{26}O_7$	8-Epiiridodial glucoside	0.00030	0.42	−0.73
220.12	14.1	$C_{10}H_{15}N_5O$	Dihydrozeatin	0.00146	0.49	−0.66

Table S3. *Cont.*

m/z	Rt	Molecular Formula	Metabolite	p-Value	Ratio D1S3/D1S1	p(corr)
271.165	5.2	$C_{13}H_{22}N_2O_4$	N-octadienoylglutamine	0.00001	0.44	−0.86
229.155	10.7	$C_{11}H_{20}N_2O_3$	Leu-Pro	0.00017	0.48	−0.79
258.17	8.1	$C_{13}H_{23}NO_4$	2-Hexenoylcarnitine	0.00175	0.49	−0.68
237.077	10.8	$C_{12}H_{14}O_5$	3-4-5-Trimethoxycinnamicacid	0.00069	0.44	−0.69
288.121	13.8	$C_{11}H_{19}N_3O_6$	Pro-Ser-Ser	0.00010	0.30	−0.72
474.231	4.5	$C_{18}H_{31}N_7O_8$	Ala-Gln-Gln-Gln	0.00002	0.44	−0.83
462.233	9.2	$C_{22}H_{31}N_5O_6$	Ala-Phe-Gln-Pro	0.00009	0.45	−0.81
218.156	13.2	$C_{11}H_{23}NOS$	Butylate	0.00029	0.51	−0.75
295.165	4.8	$C_{13}H_{22}N_5O_3$	diphthamide	0.00239	0.52	−0.64
217.155	13.2	$C_{10}H_{20}N_2O_3$	Val-Val	0.00009	0.45	−0.82
330.227	6.8	$C_{17}H_{31}NO_5$	6-Keto-decanoylcarnitine	0.00003	0.45	−0.83
358.197	10.8	$C_{16}H_{27}N_3O_6$	Glu-Ile-Pro	0.00010	0.19	−0.76
199.083	11.2	$C_7H_{10}N_4O_3$	5-Acetylamino-6-amino-3-methyluracil	0.00000	0.48	−0.84
227.103	13.6	$C_{10}H_{16}N_2O_4$	(S)-ATPA	0.00063	0.44	−0.72
216.087	14.2	$C_9H_{13}NO_5$	Succinyl proline	0.00173	0.40	−0.69
220.029	11.1	$C_7H_{11}NO_5S$	S-(3-oxo-3-carboxy-n-propyl)cysteine	0.00024	0.23	−0.75
203.139	13.5	$C_9H_{18}N_2O_3$	Leu-Ala	0.00186	0.49	−0.70
261.122	15.1	$C_{14}H_{16}N_2O_3$	Maculosin	0.00056	0.37	−0.71
304.129	8.5	$C_{15}H_{17}N_3O_4$	indole-3-acetyl-glutamine	0.00098	0.49	−0.71
233.046	11.2	$C_{12}H_{10}O_5$	2-Hydroxy-6-oxo-6-(2-hydroxyphenyl)-hexa-2,4-dienoate	0.00573	0.36	−0.58
262.038	16.0	$C_9H_{11}NSO_6$	tyrosine sulfate	0.00029	0.46	−0.78
316.212	7.2	$C_{16}H_{29}NO_5$	Butoctamide hydrogen succinate	0.00007	0.43	−0.81
174.041	15.3	$C_6H_9NO_5$	[FA amino,oxo(6:0/2:0)] 2-amino-3-oxo-hexanedioic acid	0.00130	0.50	−0.69
261.138	12.8	$C_{18}H_{16}N_2$	N,N'-Diphenyl-p-phenylenediamine	0.00049	0.51	−0.70
201.16	16.3	$C_{10}H_{20}N_2O_2$	dimethylsuberimidate	0.00075	0.43	−0.73
303.083	17.4	$C_{11}H_{16}N_2O_8$	N-Acetyl-aspartyl-glutamate	0.00514	0.38	−0.61
412.168	6.4	$C_{22}H_{25}N_3O_3S$	Ro 18-5364	0.00005	0.45	−0.82
255.097	24.5	$C_{11}H_{14}N_2O_5$	N-Ribosylnicotinamide	0.00083	0.46	−0.74
116.107	16.7	$C_6H_{13}NO$	Trimethylaminoacetone	0.00110	0.43	−0.71
188.056	13.9	$C_7H_{11}NO_5$	N-Acetyl-L-glutamate	0.00155	0.52	−0.67
266.139	8.7	$C_{14}H_{19}NO_4$	N(alpha)-Benzyloxycarbonyl-L-leucine	0.00026	0.42	−0.77
229.155	13.8	$C_{11}H_{20}N_2O_3$	Leu-Pro	0.00027	0.45	−0.78

Table S3. *Cont.*

m/z	Rt	Molecular Formula	Metabolite	*p*-Value	Ratio D1S3/D1S1	p(corr)
724.528	4.0	$C_{41}H_{74}NO_7P$	PE(18:3(6Z,9Z,12Z)/P-18:1(11Z))	0.00118	0.46	−0.70
279.064	16.0	$C_{17}H_{10}O_4$	[Fv Hydrox] 4-Hydroxyfurano[2'',3'':6,7]aurone	0.00028	0.44	−0.78
475.247	7.0	$C_{30}H_{34}O_5$	[Fv] Poinsettifolin B	0.00006	0.52	−0.79
412.23	7.4	$C_{17}H_{29}N_7O_5$	Lys-Gln-His	0.00019	0.20	−0.67
289.151	10.7	$C_{11}H_{20}N_4O_5$	Ala-Ala-Gln	0.00005	0.37	−0.82
484.31	4.4	$C_{26}H_{45}NO_5S$	[ST hydrox] N-(3alpha-hydroxy-5beta-cholan-24-oyl)-taurine	0.00771	0.53	−0.60
242.066	19.5	$C_{10}H_{11}NO_6$	N-(2,3-Dihydroxybenzoyl)-L-serine	0.00005	0.20	−0.82
228.103	14.6	$C_{14}H_{15}NO_2$	thyronamine	0.00226	0.45	−0.66
301.18	8.3	$C_{19}H_{24}O_3$	19-Oxoandrost-4-ene-3,17-dione	0.00001	0.39	−0.82
386.255	5.3	$C_{21}H_{31}N_5O_2$	Buspirone	0.00006	0.41	−0.82
296.091	16.0	$C_9H_{17}N_3O_6S$	Cys-Ser-Ser	0.00022	0.42	−0.80
221.081	11.4	$C_{12}H_{14}O_4$	[FA (12:4/2:0)] 2E,4E,8E,10E-Dodecatetraenedioic acid	0.00116	0.44	−0.69
204.052	15.0	$C_8H_{15}NOS_2$	Lipoamide	0.00189	0.46	−0.67
421.191	5.2	$C_{20}H_{28}N_4O_4S$	Leu-Trp-Cys	0.00013	0.30	−0.86
423.201	5.9	$C_{22}H_{30}O_8$	Valtratum	0.00004	0.30	−0.90
230.186	16.2	$C_{11}H_{23}N_3O_2$	N1,N8-diacetylspermidine	0.00158	0.43	−0.70
402.198	15.0	$C_{16}H_{27}N_5O_7$	Ala-Gln-Pro-Ser	0.00014	0.24	−0.75
189.076	14.8	$C_8H_{14}O_5$	(R)-3-((R)-3-Hydroxybutanoyloxy)butanoate	0.00044	0.46	−0.73
321.161	8.8	$C_{19}H_{25}O_2Cl$	11beta-Chloromethylestradiol	0.00007	0.38	−0.82
506.296	4.8	$C_{25}H_{39}N_5O_6$	Asn-Leu-Leu-Phe	0.00008	0.32	−0.81
225.088	5.1	$C_{10}H_{12}N_2O_4$	3-Hydroxy-L-kynurenine	0.00001	0.36	−0.85
418.28	8.2	$C_{21}H_{39}NO_7$	[SP amino,trihydroxy,hydroxy,methyl,oxo(20:0)] 2S-amino-3R,4R,5S-trihydroxy-2-(hydroxymethyl)-14-oxo-eicos-6E-enoic acid	0.00001	0.31	−0.84
244.058	15.8	$C_8H_{11}N_3O_6$	6-aza-uridine	0.00037	0.16	−0.76
676.32	5.0	$C_{33}H_{41}N_9O_7$	Glu-Trp-Trp-Arg	0.00425	0.48	−0.63
504.282	4.6	$C_{25}H_{37}N_5O_6$	Gln-Leu-Phe-Pro	0.00000	0.30	−0.88
399.123	15.6	$C_{25}H_{20}O_5$	[Fv methoxy,methox] (S)-2,3-Dihydro-7-methoxy-2-[2-(4-methoxyphenyl)-5-benzofuranyl]-4H-1-benzopyran-4-one	0.10096	0.42	−0.62
233.124	11.9	$C_8H_{16}N_4O_4$	(3R)-hydroxy-N-acetyl-(L)-arginine	0.00012	0.25	−0.80
454.256	9.1	$C_{20}H_{40}NO_8P$	[PC (6:0/6:0)] 1,2-dihexanoyl-sn-glycero-3-phosphocholine	0.00237	0.21	−0.72
472.209	11.0	$C_{17}H_{29}N_9O_5S$	Arg-Cys-Gly-His	0.00031	0.22	−0.68
290.16	13.0	$C_{13}H_{23}NO_6$	3-Methylglutarylcarnitine	0.00007	0.28	−0.83

Table S3. *Cont.*

m/z	Rt	Molecular Formula	Metabolite	*p*-Value	Ratio D1S3/D1S1	p(corr)
514.265	8.3	$C_{26}H_{35}N_5O_6$	Lys-Phe-Gly-Tyr	0.00005	0.28	−0.85
384.122	15.2	$C_{16}H_{21}N_3O_6S$	Phe-Asp-Cys	0.00000	0.28	−0.85
330.165	7.5	$C_{14}H_{23}N_3O_6$	Val-Asp-Pro	0.00013	0.32	−0.87
517.321	4.6	$C_{20}H_{40}N_{10}O_6$	Arg-Val-Ser-Arg	0.00012	0.31	−0.81
516.317	4.6	$C_{27}H_{41}N_5O_5$	Ile-Trp-Val-Val	0.00009	0.31	−0.86
478.277	4.7	$C_{22}H_{35}N_7O_5$	Arg-Phe-Val-Gly	0.00014	0.30	−0.82
466.251	8.2	$C_{19}H_{31}N_9O_5$	Arg-Gly-Pro-His	0.00222	0.24	−0.63
376.193	13.3	$C_{13}H_{25}N_7O_6$	Asn-Ser-Arg	0.00139	0.18	−0.70
206.102	14.2	$C_8H_{15}NO_5$	N-Acetyl-D-fucosamine	0.00007	0.24	−0.83
260.067	8.9	$C_{10}H_{14}NO_5P$	p-Nitrophenyl-O-ethyl ethylphosphonate	0.00001	0.22	−0.91
242.066	14.1	$C_{10}H_{11}NO_6$	N-(2,3-Dihydroxybenzoyl)-L-serine	0.00374	0.28	−0.62
496.276	8.8	$C_{23}H_{37}N_5O_7$	Lys-Phe-Thr-Thr	0.00082	0.12	−0.59
398.289	6.0	$C_{22}H_{39}NO_5$	[FA trihydroxy(2:0)] N-(9S,11R,15S-trihydroxy-5Z,13E-prostadienoyl)-ethanolamine	0.00046	0.24	−0.79
424.183	14.7	$C_{18}H_{25}N_5O_7$	Asn-Gln-Tyr	0.00239	0.16	−0.62
506.194	5.1	$C_{19}H_{33}N_5O_9S$	Asp-Lys-Met-Asp	0.03757	0.30	−0.56
167.038	12.6	$C_5H_{12}SO_4$	dihydroxypentenyl sulfate	0.00000	0.30	−0.78
504.266	8.9	$C_{21}H_{37}N_5O_9$	Glu-Leu-Lys-Asp	0.00037	0.28	−0.84
328.161	11.0	$C_{13}H_{21}N_5O_5$	Thr-Ala-His	0.00036	0.26	−0.62
260.115	13.7	$C_{11}H_{19}NO_6$	Lotaustralin	0.00379	0.29	−0.71
327.073	17.2	$C_{14}H_{16}O_9$	2-Succinyl-5-enolpyruvyl-6-hydroxy-3-cyclohexene-1-carboxylate	0.00157	0.30	−0.66
334.182	5.0	$C_{22}H_{25}NO_2$	Lobelanine	0.02840	0.31	−0.57
257.165	5.3	$C_{16}H_{20}N_2O$	Chanoclavine-I	0.00100	0.23	−0.72
305.045	13.2	$C_{18}H_{10}O_5$	[Fv Methy] 3',4'-Methylenedioxyfurano[2'',3'':6,7]aurone	0.00457	0.26	−0.71
357.083	18.8	$C_{15}H_{18}O_{10}$	dihydroxyphenylpropionic acid glucuronide	0.00606	0.35	−0.68
320.092	12.0	$C_{11}H_{19}N_3O_6S$	gamma-L-Glutamyl-L-cysteinyl-beta-alanine	0.00142	0.29	−0.69
490.265	5.6	$C_{24}H_{35}N_5O_6$	Ala-Leu-Thr-Trp	0.00036	0.23	−0.76
784.586	4.1	$C_{44}H_{82}NO_8P$	PC(18:2(9Z,12Z)/18:1(9Z))	0.00384	0.31	−0.65
336.164	14.5	$C_{14}H_{25}NO_8$	validoxylamine A	0.00336	0.15	−0.73
293.087	15.8	$C_{11}H_{18}O_9$	Tuliposide B	0.00635	0.28	−0.59
333.076	17.3	$C_{20}H_{14}O_5$	19-Hydroxy-8-O-methyltetrangulol	0.00684	0.09	−0.58
283.059	21.2	$C_{16}H_{10}O_5$	[Fv Methyl,hydrox] 4,5-Methylenedioxy-6-hydroxyaurone	0.01252	0.28	−0.56
554.296	5.2	$C_{29}H_{39}N_5O_6$	Gln-Leu-Phe-Phe	0.00302	0.25	−0.77

Table S3. *Cont.*

m/z	Rt	Molecular Formula	Metabolite	*p*-Value	Ratio D1S3/D1S1	p(corr)
310.071	16.0	$C_{17}H_{11}NO_5$	N-(6-Oxo-6H-dibenzo[b,d]pyran-3-yl)maleamic acid	0.00037	0.26	−0.84
328.115	12.1	$C_{11}H_{22}NO_8P$	[PC acety] 1-acetyl-2-formyl-sn-glycero-3-phosphocholine	0.00044	0.15	−0.71
260.078	17.6	$C_{10}H_{15}NO_7$	Hymexazol O-glucoside	0.00095	0.29	−0.70
534.314	5.7	$C_{24}H_{39}N_9O_5$	His-Leu-Lys-His	0.00276	0.25	−0.78
281.106	13.5	$C_{11}H_{20}O_6S$	isopropyl 6-O-acetyl-β-D-thiogalactopyranoside	0.00005	0.18	−0.77
454.139	4.1	$C_{18}H_{25}N_5O_7S$	Asn-Cys-Gly-Tyr	0.00801	0.22	−0.62
219.061	12.5	$C_7H_{12}N_2O_6$	Asp-Ser	0.00010	0.22	−0.74
556.313	5.1	$C_{29}H_{41}N_5O_6$	Lys-Phe-Val-Tyr	0.00018	0.26	−0.88
788.618	4.1	$C_{44}H_{86}NO_8P$	[PC (18:0/18:1)] 1-octadecanoyl-2-(9Z-octadecenoyl)-sn-glycero-3-phosphocholine	0.00326	0.22	−0.65
362.15	12.1	$C_{13}H_{25}N_5O_5S$	Lys-Asn-Cys	0.00181	0.18	−0.77
167.036	12.8	$C_8H_8O_4$	[PK] Orsellinic acid	0.00081	0.28	−0.74
276.017	15.9	$C_9H_9NSO_7$	dihydrodopachrome sulfate	0.00364	0.16	−0.56
338.144	14.7	$C_{13}H_{23}NO_9$	Streptobiosamine	0.00018	0.10	−0.54
237.079	13.7	$C_9H_{16}O_5S$	2-(4'-methylthio)butylmalate	0.00393	0.15	−0.61
243.1	4.0	$C_{10}H_{16}N_2O_5$	Glu-Pro	0.06619	0.34	−0.58
501.307	4.6	$C_{26}H_{44}O_9$	Mupirocin	0.00176	0.30	−0.65
309.16	7.3	$C_{19}H_{22}N_2O_2$	Sarpagine	0.05433	0.35	−0.67
259.098	4.3	$C_{15}H_{16}O_4$	[PR] Hemigossypol	0.01189	0.31	−0.65
583.312	11.5	$C_{30}H_{48}O_{11}$	Cholicacidglucuronide	0.00590	0.17	−0.56

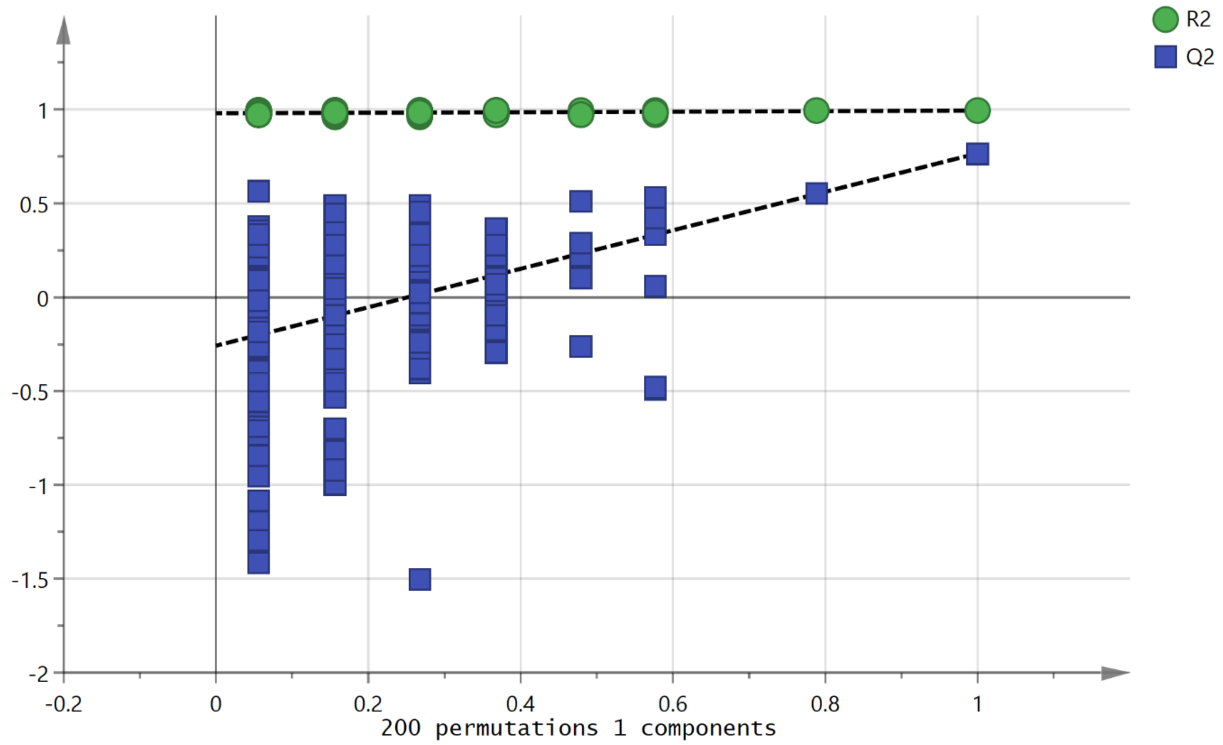

Figure S1. Cross validation model for the classification of D2S1S3 by OPLSDA.

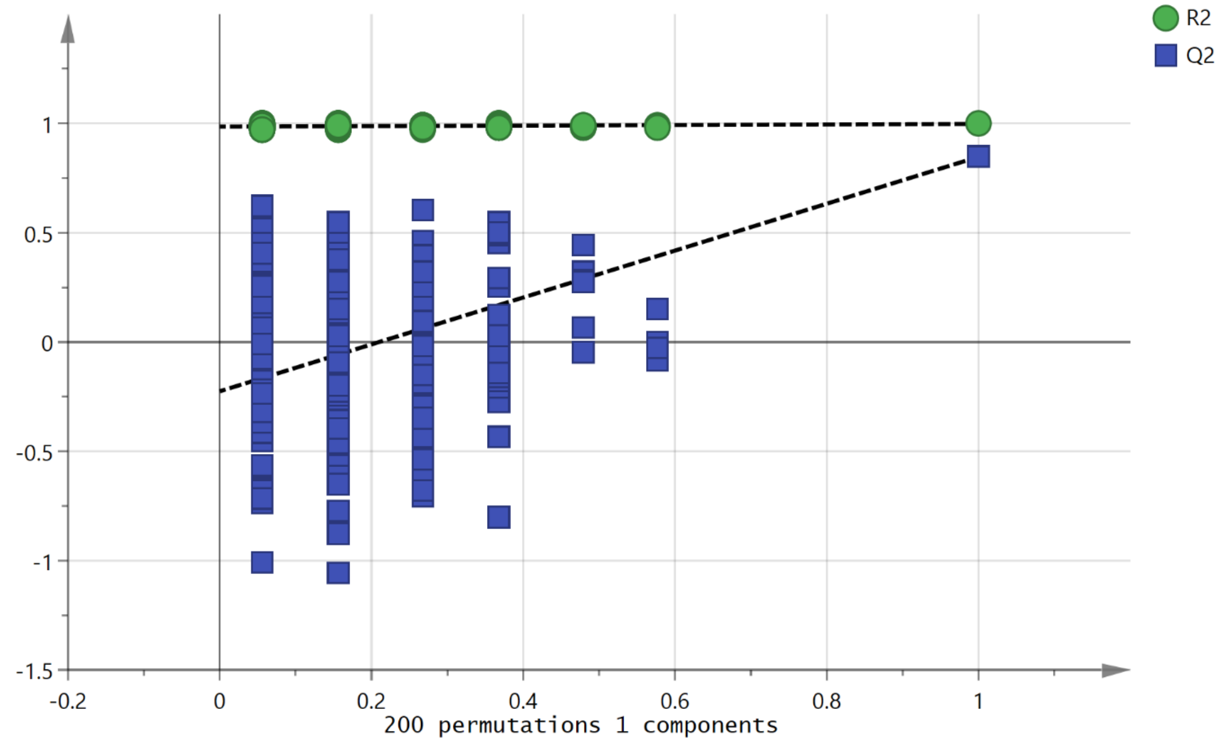

Figure S2. Cross validation of the separation of D1S1 and D1S3 by OPLSDA.

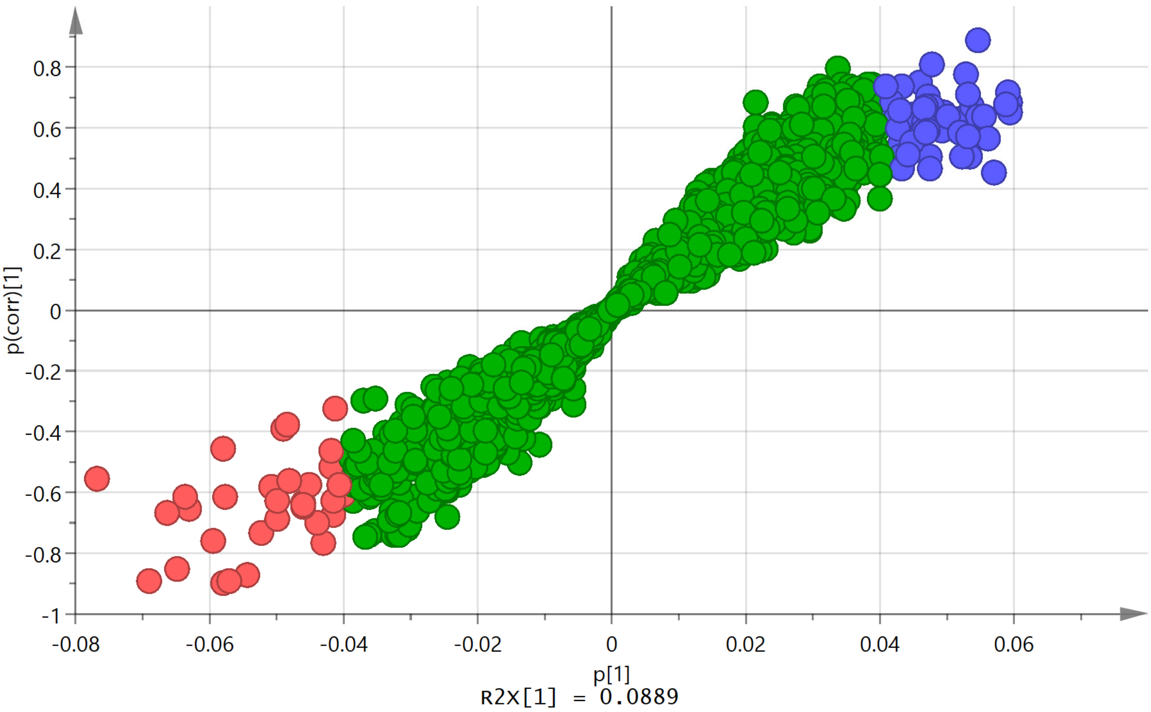

Figure S3. S-plot for OPLSDA model of D2S3 vs D2S1 highlighting the metabolites listed in Table S1.

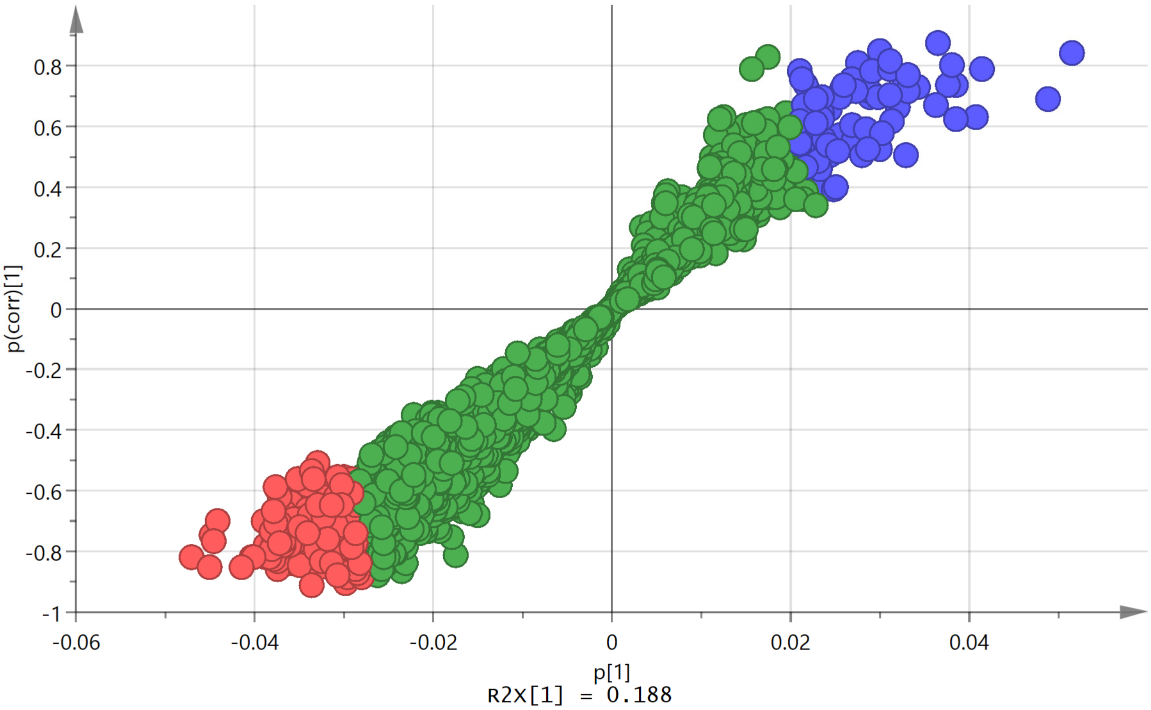

Figure S4. S-plot for OPLSDA model of D1S3 vs D1S1 highlighting the metabolites listed in Table S2.

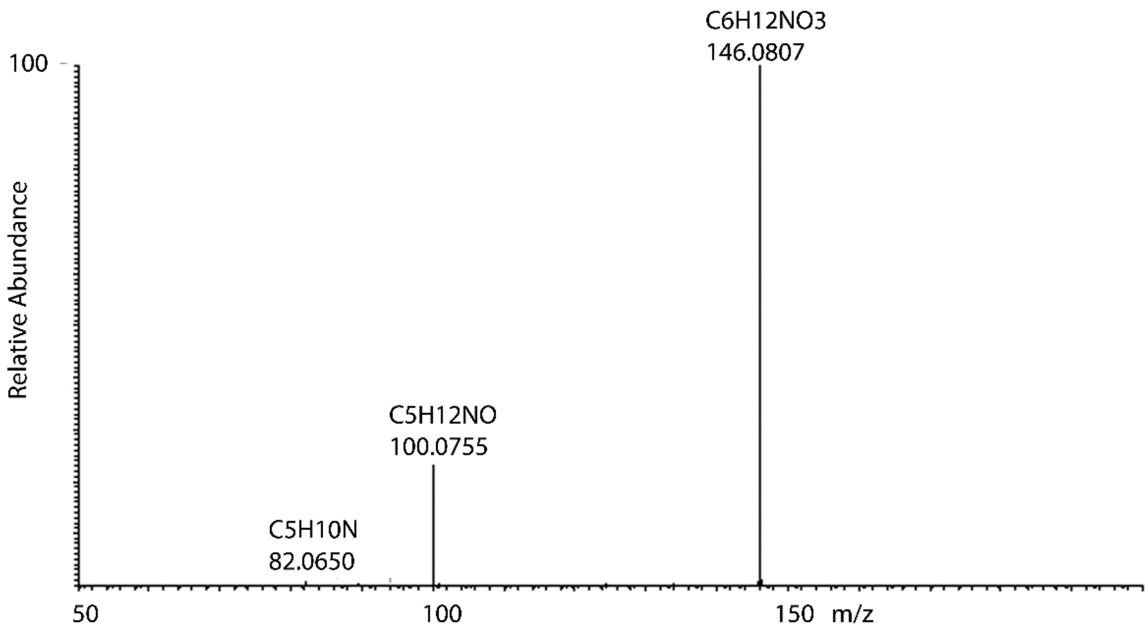

Figure S5. MS2 of oxoaminohexanoic acid obtained with a collision energy of 35V.

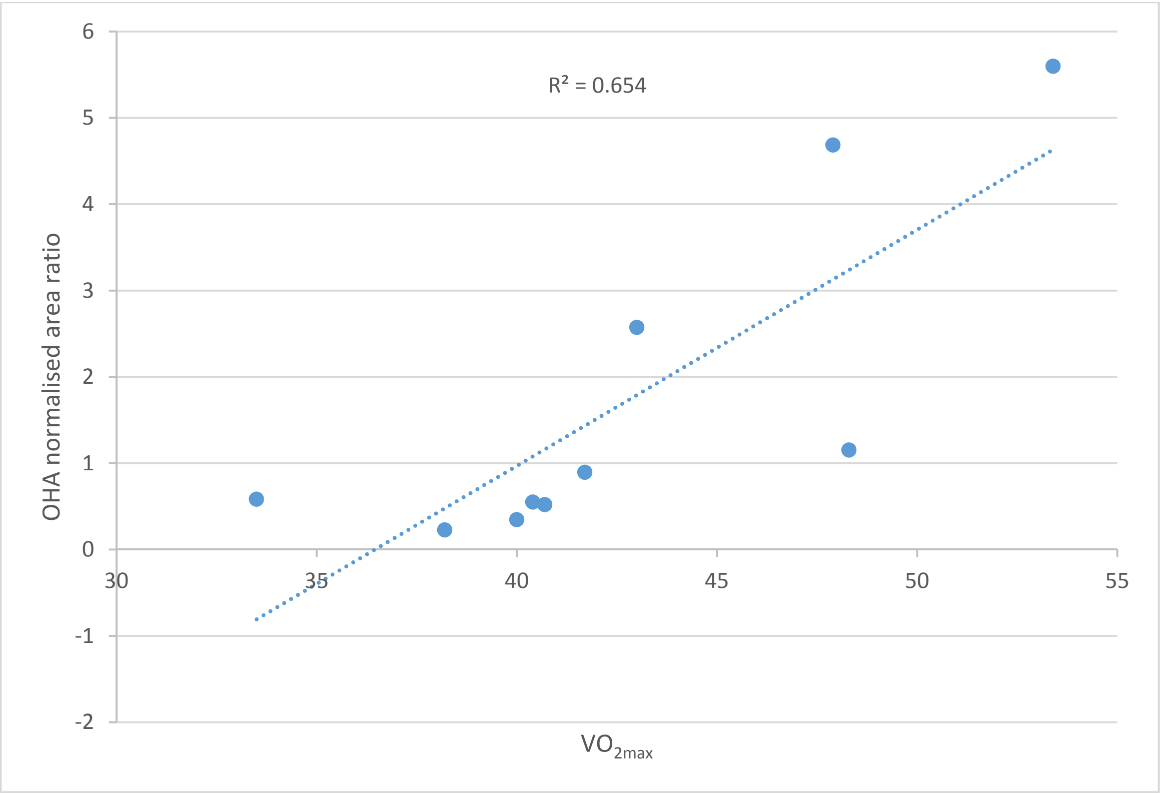

Figure S6. Plot of (area OHA D2S3/area OHA isomer D2S3)/(area OHA D2S1/area OHA isomer D2S1).
